# A finite volume adaptation of beam-to-beam contact interactions implemented for geometrically exact Simo–Reissner beams

**DOI:** 10.1007/s00466-024-02503-2

**Published:** 2024-06-11

**Authors:** Seevani Bali, Željko Tuković, Philip Cardiff, Alojz Ivanković, Vikram Pakrashi

**Affiliations:** 1 School of Mechanical and Materials Engineering, University College Dublin, Dublin, Ireland; 2 Faculty of Mechanical Engineering and Naval Architecture, University of Zagreb, Zagreb, Croatia; 3 SFI I-Form Centre, University College Dublin, Dublin, Ireland; 4 SFI MaREI Centre, University College Dublin, Dublin, Ireland; 5 UCD Centre for Mechanics, University College Dublin, Dublin, Ireland; 6 UCD Centre of Adhesion and Adhesives’, University College Dublin, Dublin, Ireland

**Keywords:** Beam-to-beam contact, Finite volume method, Frictionless, Penalty method, Augmented Lagrangian method

## Abstract

This paper presents an adaption of the finite-element based beam-to-beam contact interactions into a finite volume numerical framework. A previous work of the same authors, where a cell-centred based finite volume implementation of geometrically exact nonlinear Simo–Reissner beams was developed, is used as an underlying mathematical model. An implicit contact procedure is developed for both point-to-point and line-to-line beam frictionless contact interactions, and is implemented using the cell-centred finite volume method. To enforce the contact constraint, both penalty method and augmented-Lagrangian based techniques are used. A total of six numerical benchmark cases for point and line beam-to-beam contact interactions based on the finite element method are used to verify the numerical results, accuracy and robustness of the developed contact procedure.

## Introduction

Beam-to-beam contact interactions are extensively studied due to their relevance in engineering applications employing flexible slender structures in assemblies [1–6]. While most computational formulations for contact rely on finite element methods [7, 8], recent decades have seen finite volume-based contact approaches gain traction. Compared with the finite element techniques, the finite volume method share the same data structures and general strategy for assembling the corresponding characteristic matrices. The main conceptual difference is in the local integration domain and local integration method. For continuum formulations, this potentially allows numerical challenges to be addressed, which are yet to be fully resolved, for example, [9]: (1) spurious hourglassing and pressure checker-boarding, (2) bending difficulties, (3) shear and volumetric locking, (4) high-frequency noise in the vicinity of shocks, (5) lower order of convergence for strains and stresses in comparison with displacements, and (6) sensitivity to mesh distortions. Related to beam formulations, much less progress has been seen in finite volume formulations; however, they provide an interesting alternative that can potentially lead to improved discretisations.

Initial developments of contact formulations for steady-state and dynamic 2-D linear elastic solids are presented in Jasak et al. [10] and Tropsa et al. [11]. Later, Cardiff et al. [12, 13] extended the contact stress solver to 3-D small and large deformation problems (including friction) where the contact forces are calculated explicitly using the penalty method and are applied as traction boundary conditions. In contrast to the surface-to-surface interpolation presented in the previous references, Batistic et al. [14] developed a penalty-based segment-to-segment explicit contact force calculation method for the finite volume mechanical contact simulations and extended it to an implicit penalty-based contact algorithm for better numerical convergence in their most recent work [15]. While all the contact formulations developed in the finite volume approach are catered towards 2-D and 3-D problems, beam-to-beam contact interactions have never been adapted to the finite volume discretisation approach. This paper extends nonlinear beam contact formulations within the finite volume framework, building upon prior work implementing Simo–Reissner based geometrically exact shear-deformable beam formulation [16]. To keep the contact formulation simple and to avoid the complexities of the beam geometry in the contact procedure, rigid circular cross-section beams are assumed for contact formulation developments in this work.

Beam-to-beam contact formulations have additional kinematic constraints of the beam geometry, which differentiate them from general 3-D solid continua contact problems. The contact between beams can be point-to-point or line-to-line interaction, depending on the contact angle between the tangent vectors of the two beam centrelines at the location of contact. The contact formulation for beams with circular and rigid cross-sections and negligible shear deformations is less complex. Wriggers et al. [17, 18] presented the first frictionless and frictional point-wise contact interaction for circular cross-section beams, which was later extended to rectangular cross-sections by Litewka et al. [19]. For almost parallel beams or beams which twist around each other, line-to-line frictionless interactions are used. Overall, multiple point interactions [20] and line-to-line frictionless interactions [21–23] are used to handle different contact scenarios. Nonlinear beam formulations, including self-contact problems, are employed to demonstrate practical applications of the interaction between catenary risers and the sea bed by Neto et al. [24–26].

When the beam cross-section is assumed to be rigid and circular, the actual contact points on the beam surface can be transferred to the beam axis, and therefore, tracking the actual beam surface geometry for establishing the contact forces is not required. This is the basis of all the previously mentioned works. For non-circular and general arbitrary beam cross-sections, the analytical description for the beam surface is necessary to track the actual contact points occurring on the beam surface. Such aspects of beam contact formulations are addressed in Neto et al. [27, 28], where a master-to-master surface approach is developed and implemented for interactions between super-elliptical beam cross-sections, beam-to-surface [29], and beam-to-shell [30] interactions for both frictionless and frictional contact formulations. The above works establish a point-wise interaction between the actual surface points on the beam(s) without the bias of selecting the slave and master beams. A further extension to the surface-to-surface contact of elliptical cross-sections is made by Magliulo et al. [31, 32], where using the penalty method, the contact forces are integrated over the beams’ surfaces in contact instead of a point-wise interaction of contact points. For a smooth contact detection on the surface of the beam, the beam centroid lines are represented using Bézier curves and an additional convective coordinate in the circumferential direction of the beam cross-section is used to track the actual material points on the beam surface. A new beam-inside-beam contact formulation catering towards applications of nonlinear beam theories in the bio-medical industry using such surface-to-surface contact approach is presented in Magliulo et al. [33]. While all the previously mentioned references are based on a Simo–Reissner beam formulation and finite element numerical techniques, several authors have developed beam-to-beam contact algorithms using an alternative absolute nodal coordinate formulation [34–37].

The remainder of the paper is organised as follows. Section 2 briefly discusses the details of the beam formulation and the governing equilibrium equations, followed by the linearisation procedure. In Sect. 3, aspects of cell-centred variant of finite volume based spatial discretisation of the beam computational domain, and the discretised form of equations are discussed. Section 4 presents the contact contributions to the discretised system of equations. It further highlights the use of Hermite splines for contact detection (Sect. 4.2), the standard procedures for point-to-point (Sect. 4.4.1) and line-to-line (Sect. 4.4.2) beam contact formalism and their adaptation to the finite volume techniques. Section 4.5 presents the various contact constraint techniques implemented for the current work. Section 5 summarises the numerical solution procedure. Finally, the numerical results of point-to-point and line-to-line beam contact test cases are presented in Sect. 6. The accuracy and robustness of the current finite volume beam contact solver is tested against some well-explored contact test cases from the finite element literature.

## Beam formulation

In our recent contribution [16], a new cell-centred finite volume-based numerical formulation of geometrically nonlinear, shear-deformable Simo–Reissner beams was developed, on which the current contact framework is built. Brief details of the beam formulation, and the relevant concepts of the finite volume spatial discretisation in the context of beams are described here.

### Beam kinematics

The Simo–Reissner beam theory, initially referred to as geometrically-exact/finite-strain beam theory, is a general nonlinear beam theory which allows large 3-D deformations of the beam, including axial tension, shear, bending and torsional deformations. The initial configuration of the beam mean line is defined by a space curve $\varvec{r}_0(s) \in \mathbb {R}^3$, where $s \in [0,L]$ is the arclength parameter and *L* is the initial length of the beam. The initial cross-section orientation at arclength *s* is given by a rotation tensor $\varvec{\Lambda }_0(s)$. $\varvec{r} \equiv \varvec{r}(s,t) \in \mathbb {R}^3$ is defined as the deformed centreline of the beam space curve, which relates to the initial mean centreline curve $\varvec{r}_{0}(s)$, by $\varvec{r}(s,t) = \varvec{r}_0(s) + \varvec{w}(s,t)$, where $\varvec{w}$ is the mean displacement vector. $\varvec{\Lambda }_t(s,t)$ is the rotation tensor used to track the cross-section orientation of the beam centreline curve $\varvec{r}(s,t)$ after the deformation. Therefore, the deformed mean line $\varvec{r}(s,t)$ and the orientation of the cross section at *s* given by $\varvec{\Lambda }_t(s,t)$, fully define the deformed configuration of the beam (see, Section 2.1 of Bali et al. [16] for a detailed description).

### Governing equations

The governing equations for beam equilibrium were first presented in Simo et al. [38, 39]. These differential form of equations need to be expressed in an integral form in order to apply the finite volume based spatial discretisation to them. Therefore, an equivalent integral form of the governing (quasi-static) equations over an initial length, *L* of the beam is given by [16],

1 $$\begin{aligned}{} & {} \int _{L} \varvec{n}^\prime \ \text {d}L \ + \ \int _{L} \varvec{f} \ \text {d}L = 0 \end{aligned}$$

2 $$\begin{aligned}{} & {} \int _{L} \varvec{m}^\prime \ \text {d}L \ + \ \int _{L} (\varvec{r}^\prime \times \varvec{n}) \ \text {d} L \ + \ \int _{L} \varvec{t} \ \text {d}L = 0 \end{aligned}$$

where $\varvec{n}$ and $\varvec{m}$ are the vectors of spatial internal forces and moments acting over the beam cross-section at a certain arclength distance *s*. $\varvec{f}$ and $\varvec{t}$ are the external distributed forces and torques acting per unit reference arclength parameter *s*. The notation “$\times$" denotes the cross product between two vectors, and $(\cdot )^\prime$ operator denotes a derivative with respect to arclength parameter *s*. These spatial forces $\varvec{n}$ and moments $\varvec{m}$ can be associated with their corresponding material counterparts ($\varvec{N}$ and $\varvec{M}$) via the pull-back mapping $\varvec{\Lambda }_t^\textrm{T}$ as $\varvec{N} = \varvec{\Lambda }_t^\textrm{T}\varvec{n}$ and $\varvec{M} = \varvec{\Lambda }_t^\textrm{T}\varvec{m}$. The rotation tensor $\varvec{\Lambda }_t(s,t)$ as a push-forward (or a pull-back) operator to transform between material and spatial measures. Finally, the spatial forces $\varvec{n}$ and moments $\varvec{m}$ are related to the translational strains $\varvec{\Gamma }$ and rotational strains $\varvec{K}$ [38, 39] via the expressions, $\varvec{n} = \varvec{\Lambda }_{t} \varvec{C}_\textrm{N}\varvec{\Gamma }$ and $\varvec{m} = \varvec{\Lambda }_{t} \varvec{C}_\textrm{M}\varvec{K}$.

### Linearisation procedure

Due to the nonlinear nature of the beam kinematics and the coupled equilibrium beam equations, the spatial forces and moments must be linearised to arrive at a system of algebraic equations, which could then be iteratively solved using the Newton–Raphson algorithm. The linearised counterparts for the force $\varvec{n}$, moment $\varvec{m}$ and the coupling term $(\varvec{r}^{\prime } \times \varvec{n})$ are denoted by the operator $\text {L}[\cdot ]$. A brief derivation is provided in Appendix A, and the details can be found in Bali et al. [16]. The implicit displacement increment vector $\Delta \varvec{w}$ and rotation increment vector $\Delta \varvec{\psi }$ are the two variables for which the system of equations is solved, and these values are used to calculate the new mean line displacement vector $\varvec{w}$ and the new rotation matrix $\varvec{\Lambda }$ at the end of each Newton–Raphson iteration according to the formulae,

3 $$\begin{aligned} \varvec{w}= & {} \overset{*}{\varvec{w}} + \Delta \varvec{w} \end{aligned}$$

4 $$\begin{aligned} \varvec{\Lambda }= & {} \exp (\widehat{\Delta \varvec{\psi }}) \overset{*}{\varvec{\Lambda }} \end{aligned}$$

where superscript notation $\overset{*}{(\cdot )}$ denotes the numerical values evaluated in the previous Newton–Raphson iteration, and the $\widehat{(\cdot )}$ operator denotes a skew-symmetric matrix associated with the corresponding (pseudo)-vector given by the relation, $\varvec{a} \times \varvec{h} = \widehat{\varvec{a}} \varvec{h} \ \forall \ \varvec{h} \in \mathbb {R}^3$. The exponentiation of the skew-symmetric tensor $\widehat{\Delta \varvec{\psi }}$ in Eq. 4 is evaluated by substituting the incremental rotation vector $\Delta \varvec{\psi }$ in the Rodrigues’ formula (given in Eq. 12 in Bali et al. [16]) to compute the rotation matrix $\varvec{\Lambda }$.

## Finite volume numerical model

In this section, cell-centred FV discretisation of the computational domain and the governing balance equations is summarised.

### Spatial discretisation of beam domain

**Fig. 1:**
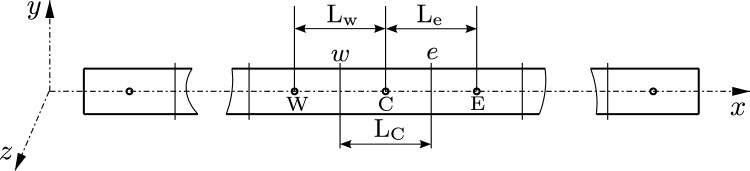
Beam body in reference configuration discretised by a finite set of 1-D CVs (cells). Image directly adapted from Bali et al. [16]

The beam body, when in its initial state, is divided into a finite number of uniform sections or control volumes (CVs), as depicted in Fig. 1. The computational stencil, illustrated in Fig. 1, comprises the central CV (cell) with a length of $\text {L}_{\text {C}}$ and a computational node $\text {C}$ positioned at the cell centroid. This central CV is bounded by two internal faces, denoted as *w* and *e*, which are shared with the neighbouring west and east cells. The neighbouring cells have centroids at $\text {W}$ and $\text {E}$ respectively, and lengths of $\text {L}_{\text {w}}$ and $\text {L}_{\text {e}}$ measured from the node $\text {C}$.

**Fig. 2:**
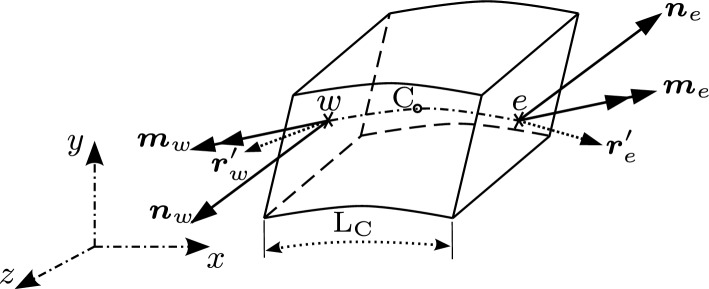
Balance of forces and moments on an isolated CV in the deformed configuration. Image directly adapted from Bali et al. [16]

### Equation discretisation

For an isolated CV in the deformed configuration of the beam (Fig. 2), the integral form of the balance equations (Eqs. 1 and 2) can be discretised over a CV as following,

5 $$\begin{aligned}&\int _{\text {L}_{\text {C}}} \varvec{n}^\prime \text {d}L = \varvec{n} \big |_{w}^{e} = \varvec{n}_{e} - \varvec{n}_{w} \quad ; \quad \int _{\text {L}_{\text {C}}} \varvec{f} \ \text {d}L \approx \varvec{f}_{\text {C}} \text {L}_{\text {C}} \hspace{0.2cm} \nonumber \\&\qquad \implies \varvec{n}_{e} - \varvec{n}_{w} + \varvec{f}_{\text {C}} \text {L}_{\text {C}} = 0 \end{aligned}$$

where $\varvec{n}_{e}$ and $\varvec{n}_{w}$ are the force values evaluated at cell faces *e* and *w* respectively and the subscript $\text {C}$ represents the values at cell-centre $\text {C}$. The term $\varvec{f}$ is assumed to have a linear variation across the CV and hence, can be approximated by the mid-point rule.

The moment balance equation is discretised about the cell-centre $\text {C}$. The first term of the moment balance equation can be exactly evaluated at cell boundaries, and the integral of $(\varvec{r}^\prime \times \varvec{n})$ is approximated over the CV using the trapezoidal rule and is evaluated at the faces *w* and *e* respectively. The final discretised moment equation takes the form,

6 $$\begin{aligned} \begin{aligned}&\int _{\text {L}_{\text {C}}} \varvec{m}^\prime \text {d} L = \varvec{m} \big |_{w}^{e} = \varvec{m}_{e} - \varvec{m}_{w}; \hspace{0.2cm} \int _{\text {L}_{\text {C}}} (\varvec{r}^\prime \times \varvec{n}) \ \text {d} L \\&\quad = \frac{1}{2}\text {L}_{\text {C}}(\varvec{r}^\prime _{e} \times \varvec{n}_{e})+ \frac{1}{2}\text {L}_{\text {C}}(\varvec{r}^\prime _{w} \times \varvec{n}_{w}) \\&\quad \implies \varvec{m}_{e} - \varvec{m}_{w} + \frac{1}{2}\text {L}_{\text {C}}(\varvec{r}^\prime _{e} \times \varvec{n}_{e}) + \frac{1}{2}\text {L}_{\text {C}}(\varvec{r}^\prime _{w} \times \varvec{n}_{w}) \\&\qquad + \varvec{t}_{\text {C}} \text {L}_{\text {C}} = 0 \end{aligned} \end{aligned}$$

Here, $\varvec{t}$ is also assumed to have a linear variation across the CV and hence is approximated by the mid-point rule.

The discretised equations (Eqs. 5,6) are substituted with the corresponding linearised expressions (Appendix A) and are evaluated at the cell-faces *w* and *e*. The mean line displacement correction vector $\Delta \varvec{w}$ and its derivative $\Delta \varvec{w}^\prime$, along with the rotational correction vector $\Delta \varvec{\psi }$ and its derivative $\Delta \varvec{\psi }^\prime$ are approximated at the face centres using central finite difference scheme for derivatives and linear interpolation for cell-face values. The face-centre values on the internal faces *w* and *e* are interpolated using cell-centre values as,

7 $$\begin{aligned} \Big [(\cdot ) \Big ]_{f} = \gamma _{f} \Big [(\cdot ) \Big ]_{\text {N}} + (1 - \gamma _{f}) \Big [(\cdot ) \Big ]_{\text {C}} \end{aligned}$$

where subscript ‘$\text {N}$’ refers to either east or west neighbour cell-centre value. $\gamma _f = \frac{1}{2} \frac{\text {L}_{\text {C}}}{\text {L}_f}$ is the weighting factor, where $\text {L}_f$ is the length $\text {L}_\text {w}$ or $\text {L}_\text {e}$ depending on the required face centre value. The cell face derivatives at the internal faces $\mathrm w$ and $\mathrm e$ given by $\Big [(\cdot ) \Big ]^\prime _{e}$ and $\Big [(\cdot ) \Big ]^\prime _{w}$ are approximated using the central finite difference scheme as,

8 $$\begin{aligned} \Big [(\cdot ) \Big ]^\prime _{e} = \frac{\Big [(\cdot ) \Big ]_{\text {E}} - \Big [(\cdot ) \Big ]_{\text {C}} }{\text {L}_{\text {e}}} \quad ; \quad \Big [(\cdot ) \Big ]^\prime _{w} = \frac{\Big [(\cdot ) \Big ]_{\text {C}} - \Big [(\cdot ) \Big ]_{\text {W}} }{\text {L}_{\text {w}}} \end{aligned}$$

In the discretised equilibrium equations, all the terms are *explicitly* computed except the unknowns, incremental displacement and incremental rotation ($\Delta \varvec{w}$ and $\Delta \varvec{\psi }$) vectors, which are treated *implicitly* and are evaluated at the computational cell-centres. At the end of every Newton-Raphson iteration, $\Delta \varvec{w}$ and $\Delta \varvec{\psi }$ are used to update the deformed mean line position vector $\varvec{r}(s)$ and the new rotation matrix $\varvec{\Lambda }$ according to the Eqs. 3 and 4.

## Contact formulation

This section outlines the details of the finite volume contact procedure adapted for the beam-to-beam contact interactions.

### Discretised equations including contact

The contact forces acting on the beam body are applied implicitly using a Newton–Raphson iterative scheme. The total nonlinear contact force ($\varvec{n}_c$) acting at an arbitrary distance ${\textrm{d}}\varvec{r}$ from the cell-centre $\textrm{C}$ of the beam CV is converted into a force acting at $\textrm{C}$ and an equivalent contact moment about the cell-centre $\textrm{C}$ due to the skewness of the contact force, i.e., $\varvec{m}_c= {\textrm{d}}\varvec{r} \times \varvec{n}_c$. To that end, the discretised form of the (integral) balance equations Eq. 1 and Eq. 2 for an isolated CV, including the contact forces, can be expressed as follows,

9a $$\begin{aligned}&\varvec{n}_{e} - \varvec{n}_{w} + \varvec{f}_{\text {C}} \text {L}_{\text {C}} + \varvec{n}_c = 0 \end{aligned}$$

9b $$\begin{aligned}&\varvec{m}_{e} - \varvec{m}_{w} + \frac{1}{2}\text {L}_{\text {C}}(\varvec{r}^\prime _{e} \times \varvec{n}_{e}) + \frac{1}{2}\text {L}_{\text {C}}(\varvec{r}^\prime _{w} \times \varvec{n}_{w}) \nonumber \\&\qquad + \varvec{t}_{\text {C}} \text {L}_{\text {C}} + \varvec{m}_c= 0 \end{aligned}$$

### Hermite spline interpolation

The highest derivative present in the equilibrium equations of the Simo-Reissner beams is the first-order arclength derivative of the beam centreline $\varvec{r}^{\prime }(s)$, which is computed using the central finite difference scheme (Eq. 8) is discontinuous. Using such discontinuous beam centreline derivatives for the contact procedure might lead to numerical convergence issues and oscillations in the contact force distribution. A continuous and smooth representation of beam centreline curves is necessary for the contact formulation [21, 40] to ensure better detection of contact locations and to avoid discontinuous jumps in the contact forces, especially for frictional and sliding contact problems. Hence, a $C^1-$continuous Hermite spline interpolating polynomials for the beam-to-beam contact detection is used in this work, details of which are provided in Appendix B.

The Hermite spline interpolation is used only to detect and update the location of contact points in the beam and not in the discretised balance equations of the beam body. More specifically, for calculating the contact moment $\varvec{m}_c = {\textrm{d}}\varvec{r} \times \varvec{n}_c$, the term ${\textrm{d}}\varvec{r}$ is calculated following the cell-centred finite volume spatial discretisation principles instead of using Hermite spline polynomials. Therefore, the contact moment $\varvec{m}_c$ can be expanded as,

10 $$\begin{aligned} \varvec{m}_c \equiv {\textrm{d}} \varvec{r} \times \varvec{n}_c&= (\varvec{r}^\prime _{c} \ {\textrm{d}} s) \times \varvec{n}_c \end{aligned}$$

11 $$\begin{aligned}&= \frac{\text {L}_{\text {C}}}{2} \ \xi _c \ (\varvec{r}^\prime _{c} \times \varvec{n}_c) \end{aligned}$$

where the term $\varvec{r}^\prime _{c}$ at the moment equation represents the arclength derivative of the deformed centreline at the contact point $\xi _c$ given by,

12 $$\begin{aligned} \varvec{r}^\prime _c = {\left\{ \begin{array}{ll} \varvec{r}^\prime _{e}, &{} \xi _c > 0 \\ \varvec{r}^\prime _{w}, &{} \xi _c < 0 \end{array}\right. } \end{aligned}$$

Here, the terms $\varvec{r}^\prime _e$ or $\varvec{r}^\prime _w$ in Eq. 10 are calculated using central-difference scheme (Eq. 8) instead of using Hermite spline derivatives. Such an assumption of the arclength derivative $\varvec{r}^\prime _c$ results in a mesh discretisation error in the equations, which can be reduced by refining the mesh. Furthermore, when $\xi _c = 0$, there is no moment contribution due to the contact force $\varvec{n}_c$ at the cell centre $\text {C}$ of the isolated CV.

### Contact conditions

Contact is usually solved as a constrained optimisation problem, where the following impenetrability constraints (also known as Karush Kuhn-Tucker (KKT) conditions) are imposed,

13a $$\begin{aligned} g_n&\ge 0 \end{aligned}$$

13b $$\begin{aligned} n_c&\le 0 \end{aligned}$$

13c $$\begin{aligned} n_c \cdot g_n&= 0 \end{aligned}$$

The first condition Eq. 13a, states a no penetration condition where the normal distance $g_n$ between two opposite bodies in contact may either be positive or zero. The second Eq. 13b is the condition for contact force to be either zero or compressive, and the last Eq. 13c presents a consistency condition stating that for a positive gap (no contact), contact forces cannot occur.

### Types of beam-to-beam contact formulation

To calculate the normal (frictionless) contact forces acting on the beam(s), two different beam-to-beam contact formulations, i.e., (a) point-to-point contacts for large contact angles between beams and (b) line-to-line contact formulations for almost parallel beams is described in the following two sections.

#### Point-to-point beam contact

**Fig. 3:**
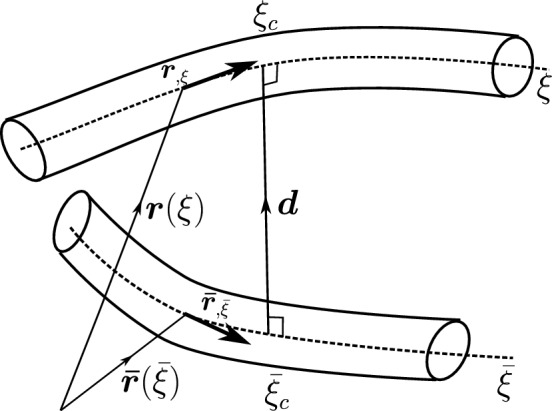
Point-to-point contact of beams: a minimal distance problem with two orthogonality conditions

This section presents a standard point-to-point beam contact formulation introduced by Zavarise et al. [17]. Two arbitrary beams with circular cross-sections of radii $R_1$ and $R_2$ are considered. The beam centrelines are represented by $\varvec{r}(\xi )$ and $\bar{\varvec{r}}(\bar{\xi })$ (Fig. 3), where $\xi$ and $\bar{\xi }$ are the local parametric coordinates of the two respective beams. In the point contact formulation, the beams are assumed to be in contact at a single point. The contact constraint is enforced at the closest projection points on the beam centrelines, given by $\xi _c$ and $\bar{\xi }_c$ (Fig. 3). The contact point-pair ($\xi _c$, $\bar{\xi }_c$) is determined by solving a minimal distance problem between two beam centrelines with the condition that the minimal distance vector ($\varvec{d}_p$) is orthogonal with the beam centrelines at the locations $\xi _c$ and $\bar{\xi }_c$ respectively (Eq. 14).

14 $$\begin{aligned} \varvec{d}_p := \min _{\xi ,\bar{\xi }} d(\xi ,\bar{\xi }) = d(\xi _c,\bar{\xi }_c) \end{aligned}$$

Here, $d(\xi ,\bar{\xi }) = ||\varvec{r}(\xi ) - \varvec{\bar{\varvec{r}}}(\bar{\xi })||$. This condition leads to the minimisation of two orthogonality conditions $(q_1,q_2)$ given by,

15a $$\begin{aligned} q_1(\xi , \bar{\xi })&\equiv \left[ \varvec{r}(\xi ) - \bar{\varvec{r}}(\bar{\xi }) \right] \cdot \varvec{r}_{,\xi } = 0 \end{aligned}$$

15b $$\begin{aligned} q_2(\xi , \bar{\xi })&\equiv \left[ \varvec{r}(\xi ) - \bar{\varvec{r}}(\bar{\xi })\right] \cdot \bar{\varvec{r}}_{,\bar{\xi }} = 0 \end{aligned}$$

Depending on the polynomial functions used to represent the beam centrelines, $\varvec{r}_c$ and $\bar{\varvec{r}}_c$, the orthogonality equations (Eqs. 15) can either be linear or nonlinear functions of $\xi$ and $\bar{\xi }$. For an assumption of Hermite spline interpolation of the beam centrelines, these equations are nonlinear; therefore, an iterative procedure is required to estimate the actual contact pair ($\xi _c$, $\bar{\xi }_c$). Details on solving for a possible contact pair and the contact search algorithm implemented is outlined in Appendix C.1. Assuming that the location of the contact pair ($\xi _c$, $\bar{\xi }_c$) is known, contact forces of identical absolute value but opposite sign are applied at that location when the penetration condition (Eq. 13a) is violated. The (normal) contact force is given by,

16 $$\begin{aligned} \varvec{n}_c = {\left\{ \begin{array}{ll} - \ p_{n} \ g_{n} \varvec{d}, &{} \text {if}\ g_{n} \le 0 \\ 0, &{} \text {if}\ g_{n} > 0 \end{array}\right. } \end{aligned}$$

where $\varvec{n}_c$ is the frictionless contact force contribution, $p_n$ is the penalty stiffness for normal contact, $g_n$ is the magnitude of the normal gap vector between the two beams, and $\varvec{d}$ is the unit distance vector along the direction of the gap. The equations for $g_n$ and $\varvec{d}$ are given by,

17 $$\begin{aligned} g_n= & {} || \varvec{r} (\xi _c) - \bar{\varvec{r}}(\bar{\xi }_c) || - (R_1 + R_2) \end{aligned}$$

18 $$\begin{aligned} \varvec{d}= & {} \frac{ \varvec{r} (\xi _c) - \bar{\varvec{r}}(\bar{\xi }_c)}{||\varvec{r} (\xi _c) - \bar{\varvec{r}}(\bar{\xi }_c)||} \end{aligned}$$

The contact force (Eq. 16) is in general nonlinear and can be linearised about the contact force in the previous Newton–Raphson iteration ($\overset{*}{\varvec{n}}_{c}$) as follows,

19 $$\begin{aligned} \textrm{L}[\varvec{n}_{c}]&= \overset{*}{\varvec{n}}_{c} + \left( \frac{\partial n_c}{\partial g_{n}} \right) \Delta g_n(\xi ,\bar{\xi }) \ \varvec{d} + n_c \ \Delta \varvec{d}(\xi ,\bar{\xi }) \end{aligned}$$

where $n_c = - p_n g_n$ is the magnitude of contact force, $\Delta g_n(\xi ,\bar{\xi })$ and $\Delta \varvec{d}(\xi ,\bar{\xi })$ are the linearised quantities of $g_n$ and $\varvec{d}$ respectively. Following the derivation presented in Wriggers et al., [17], these linearised forms can be expressed as,

20a $$\begin{aligned} \Delta g_n(\xi ,\bar{\xi })&= \big ( \Delta \varvec{r}(\xi ) - \Delta \bar{\varvec{r}}(\bar{\xi }) \big ) \cdot \varvec{d} \end{aligned}$$

20b $$\begin{aligned} \Delta \varvec{d}(\xi ,\bar{\xi })&= \big ( {\textbf {I}} - \varvec{d} \otimes \varvec{d} \big ) \cdot \big ( \Delta \varvec{r}(\xi ) - \Delta \bar{\varvec{r}}(\bar{\xi }) \big ) \end{aligned}$$

where the terms $\Delta \varvec{r}(\xi )$ and $\Delta \bar{\varvec{r}}(\bar{\xi })$ are the linearised form of the deformed beam centrelines. The contact formulation is deformation-dependent because, after every Newton–Raphson iteration, the beam’s geometry changes; therefore, the previously detected point of contact is also subjected to change. In a particular iteration, the correction terms $\Delta \varvec{r}(\xi )$ and $\Delta \bar{\varvec{r}}(\bar{\xi })$ depend on the incremental changes in the detected contact pair ($\Delta \xi _c$, $\Delta \bar{\xi }_c$) and the displacement correction vectors of both the beams in contact ($\Delta \varvec{w}$, $\Delta \bar{\varvec{w}}$). The appropriate linearised form of the deformed beam centrelines is given by,

21a $$\begin{aligned} \Delta \varvec{r}(\xi )&= \varvec{r}_{,\xi } \Delta \xi _c + \Delta \varvec{w} \end{aligned}$$

21b $$\begin{aligned} \Delta \bar{\varvec{r}}(\bar{\xi })&= \bar{\varvec{r}}_{,\bar{\xi }} \Delta \bar{\xi }_c + \Delta \bar{\varvec{w}} \end{aligned}$$

**Fig. 4:**
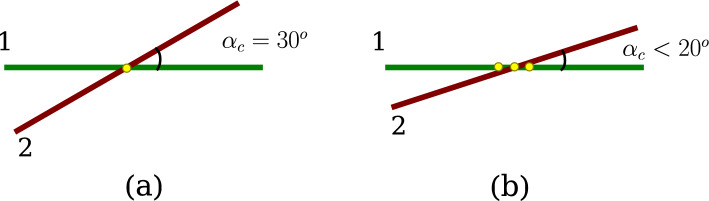
Contact angle between beam centrelines: **a** $\alpha _c = 30^{\circ }$, a unique point of contact exists, **b** $\alpha _c < 20^{\circ }$, multiple possibilities of point contacts

where the first-order derivatives $\varvec{r}_{,\xi }$ and $\bar{\varvec{r}}_{,\bar{\xi }}$ are evaluated at the contact point-pair $(\xi _c, \bar{\xi }_c)$ using the Hermite spline polynomials (Appendix B). The values of $\Delta \xi _c$ and $\Delta \bar{\xi }_c$ are obtained from the linearisation of the two orthogonality conditions $q_1$ and $q_2$ about the contact point pair $(\xi _c, \bar{\xi }_c)$ [17] as given by,

22 $$\begin{aligned}{} & {} {[}M] \begin{bmatrix} \Delta \xi _c \\ \Delta \bar{\xi }_c \end{bmatrix} =\begin{bmatrix} -\bar{\varvec{r}}^\textrm{T}_{,\bar{\xi }} &{} \quad \bar{\varvec{r}}^\textrm{T}_{,\bar{\xi }} \\ -\varvec{r}^\textrm{T}_{,\xi } &{} \quad \varvec{r}^\textrm{T}_{,\xi } \end{bmatrix} \begin{bmatrix} \Delta \varvec{w} \\ \Delta \bar{\varvec{w}} \end{bmatrix}\nonumber \\{} & {} \qquad \qquad \qquad \quad +\begin{bmatrix} \textbf{0} &{} -(\varvec{r} - \bar{\varvec{r}})^\textrm{T} \\ -(\varvec{r} - \bar{\varvec{r}})^\textrm{T} &{} \textbf{0} \end{bmatrix} \begin{bmatrix} \Delta \varvec{w}_{,\xi } \\ \Delta \bar{\varvec{w}}_{,\bar{\xi }} \end{bmatrix} \end{aligned}$$

where

23 $$\begin{aligned} {[}M] =\begin{bmatrix} \varvec{r}_{,\xi } \cdot \varvec{\bar{\varvec{r}}}_{,\bar{\xi }} &{} \left( \varvec{r} - \bar{\varvec{r}}\right) \cdot \bar{\varvec{r}}_{,\bar{\xi }\bar{\xi }} - \bar{\varvec{r}}_{,\bar{\xi }} \cdot \bar{\varvec{r}}_{,\bar{\xi }}\\ \left( \varvec{r} - \bar{\varvec{r}}\right) \cdot \varvec{r}_{,\xi \xi } + \varvec{r}_{,\xi } \cdot \varvec{r}_{,\xi } &{} -\bar{\varvec{r}}_{,\bar{\xi }} \cdot \varvec{r}_{,\xi } \end{bmatrix} \end{aligned}$$

For the current FV contact formulation between beams, the displacement corrections $\Delta \varvec{w}$ and $\Delta \bar{\varvec{w}}$ are assumed to be constant for a CV, and hence the values $\Delta \varvec{w}_{,\xi } = \Delta \bar{\varvec{w}}_{,\bar{\xi }} = 0$. Using the Eqs. 22 and 21 in the Eqs. 20, the linearised form of the contact force Eq. 19 can be evaluated.

The point-to-point contact beam formulation is sufficient when the contact angle $\alpha _c$ between the beam centrelines is sufficiently large (Fig. 4). However, for a small range of angles and almost parallel beam configuration, estimating a unique point satisfying both the orthogonality conditions (Eq 15) is not possible. The lower limit of contact angles beyond which the point formulation will fail is hard to predict. Meier et al. [21] presented a heuristic way to estimate it, along with developing an all-angle beam contact formulation [41] where a smooth transition algorithm between point and line contact formulation. The implementation of such transitioning algorithms will be explored in the future. A general rule of thumb is that the point contact formulation fails when the contact angles between two beams fall below $20^{\circ }$. For such scenarios, a line-to-line contact formulation between beams is required; the details of this are provided next.

#### Line-to-line beam contact formulation

**Fig. 5:**
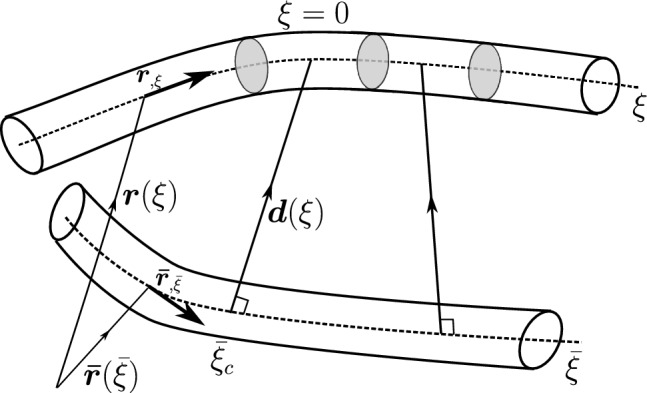
Line-to-line contact interaction between beams

The line-to-line contact formulation, first implemented by Meier et al. [21], is described here. The double orthogonality condition of the point contact formulation becomes over-constrained when the beams are almost parallel in configuration. Hence, an estimation of unique contact pair $(\xi _c, \bar{\xi }_c)$ on both beams is not possible. To overcome this problem, the constraint on one side of the beam is released, which results in a single orthogonality condition on the neighbouring beam with centreline $\varvec{\bar{\varvec{r}}}(\bar{\xi })$ as follows (Fig. 5),

24 $$\begin{aligned} \varvec{d}_l(\xi ):= \min _{\bar{\xi }} d(\xi ,\bar{\xi }) = d(\xi ,\bar{\xi }_c) \end{aligned}$$

Here, $\varvec{d}_l(\xi ) = ||\varvec{r}(\xi ) - \varvec{\bar{\varvec{r}}}(\bar{\xi })||$ is the distance vector between two beam centrelines. From a chosen $\xi$ coordinate on the first beam (slave), an orthogonal distance vector to the nearest point $\bar{\xi }_c$ on the neighbouring beam (master) is projected. For a chosen $\xi$, the orthogonality condition ($q_2(\xi ,\bar{\xi })$) which needs to be solved is given by (Fig. 5),

25 $$\begin{aligned} q_2(\xi , \bar{\xi }) \equiv \left[ \varvec{r}(\xi ) - \bar{\varvec{r}}(\bar{\xi })\right] \cdot \bar{\varvec{r}}_{,\bar{\xi }} = 0 \end{aligned}$$

The corresponding contact force due to the line-to-line interaction between beams can be expressed as,26where $\varvec{n}_c$ is the integral of the line contact force distribution acting at different locations $\xi$ across the length of the beam CV. Also, $g_n(\xi )$ is the magnitude of the gap vector between the two beams, and $\varvec{d}(\xi )$ is the unit distance vector along the gap function. The equation for $g_n(\xi )$ is given by,

27 $$\begin{aligned} g_n (\xi ) = \Vert \varvec{r}(\xi ) - \varvec{\bar{\varvec{r}}}(\bar{\xi }_c) \Vert - (R_1 + R_2) \end{aligned}$$

##### Remark

In Eq. 27, the gap function calculated by subtracting the cross-section radii directly from the magnitude of the distance vector $\varvec{d}_l(\xi )$ results in a geometrical error which stems from the fact that the distance vector $\varvec{d}_l(\xi )$ is not perpendicular to the beam centreline $\varvec{r}$. This error is acceptable for generally thin beams whose cross-section radius is much smaller than their length (high slenderness ratio).

Unlike the unit distance vector $\varvec{d}$ in Eq. 18 in point contact formulation which is estimated for a detected contact pair of points ($\xi _c$, $\bar{\xi }_c$), the unit distance vector $\varvec{d} (\xi )$ for line contact is a function of the contact point $\xi$ as given by,

28 $$\begin{aligned} \varvec{d} (\xi ) = \frac{\varvec{r}(\xi ) - \varvec{\bar{\varvec{r}}}(\bar{\xi }_c)}{\Vert \varvec{r}(\xi ) - \varvec{\bar{\varvec{r}}}(\bar{\xi }_c) \Vert } \end{aligned}$$

The contact force (Eq. 26) is in general nonlinear and can be linearised about the contact force in the previous Newton–Raphson iteration ($\overset{*}{\varvec{n}}_{c}$) as follows,

29 $$\begin{aligned} \textrm{L}[\varvec{n}_{c}]&= \overset{*}{\varvec{n}}_{c} + \left( \frac{\partial n_c}{\partial g_{n}} \right) \Delta g_n(\bar{\xi }) \ \varvec{d} (\xi ) + n_c \ \Delta \varvec{d}(\bar{\xi }) \end{aligned}$$

where $\Delta g_n(\bar{\xi })$ and $\Delta \varvec{d}(\bar{\xi })$ are the linearised quantities of $g_n$ and $\varvec{d}$ respectively. These quantities are derived from Eqs. 20, where a known and chosen $\xi$ location on the slave beam renders the variation of $\Delta \xi =0$. Thus, the variation of slave beam centreline (Eq. 21a) $\Delta \varvec{r}(\xi ) = \Delta \varvec{w}$. Therefore, the linearised expressions for $\Delta g_n(\bar{\xi })$ and $\Delta \varvec{d}(\bar{\xi })$ are functions of $\bar{\xi }$, and are given by,

30a $$\begin{aligned} \Delta g_n(\bar{\xi })&= \big ( \Delta \varvec{w} - \Delta \bar{\varvec{r}}(\bar{\xi }) \big ) \cdot \varvec{d} \end{aligned}$$

30b $$\begin{aligned} \Delta \varvec{d}(\bar{\xi })&= \big ( {\textbf {I}} - \varvec{d} \otimes \varvec{d} \big ) \cdot \big ( \Delta \varvec{w} - \Delta \bar{\varvec{r}}(\bar{\xi }) \big ) \end{aligned}$$

Here, $\Delta \bar{\varvec{r}}(\bar{\xi })=\bar{\varvec{r}}_{,\bar{\xi }} \Delta \bar{\xi }_c + \Delta \bar{\varvec{w}}$. For every Newton–Raphson iteration, the $\Delta \bar{\xi }_c$ value is calculated using the linearised form of the orthogonality equation (Eq. 25). Using the $\Delta \bar{\xi }_c$ value along with the Eq. 30, the linearised contact force, Eq. 29 is computed for every iteration.

In a conventional FE framework, the line integral for contact force calculation (Eq. 26) is approximated as a summation of the contact forces computed for different locations of $\xi$ on the slave beam where the locations of $\xi$ depend on the user-specified number of Gauss integration points. However, for the current FV line contact formulation, only one Gauss-integration point located at the cell centre of a beam CV ($\xi =0$) is used to evaluate the integral line force distributed over the isolated CV length. Hence, one could denote the contact force $n_c$ in Eq. 26 as discrete line contact force. Naturally, such line contact formulation depends on the spatial discretisation of the beam domain, and the error in the force calculations can be reduced for a finer mesh discretisation. Additionally, for the line contact search procedure, a two-half pass contact procedure is adopted [31, 42], whose details are discussed in Appendix C.2.

### Contact constraints methods

Amongst various methods to impose contact constraints, the three major ones are the penalty approach, Lagrange multipliers technique, and the augmented Lagrangian method (ALM) [7]. In the current work, the penalty and ALM contact constraint methods are implemented for beam-to-beam contact.

#### Penalty method

During the contact simulation between two beams, the penetration between beams is checked after every iteration. Ideally, a zero penetration is desired (KKT conditions in Eq. 13). To enforce the contact constraint, a penalty law is considered, which aims to minimise the violation of the penetration constraint. Accordingly the contact force is calculated as,

31 $$\begin{aligned} n_c(g_n) = {\left\{ \begin{array}{ll} - p_{n} \ g_{n}, &{} g_{n} \le 0 \\ 0, &{} g_{n} > 0 \end{array}\right. } \end{aligned}$$

where $p_n$ is the penalty stiffness value, and $g_n$, is the amount of penetration occurring at the contact location. The contact force in Eq. 31 is based on a linear penalty law, which leads to a kink in the force graph due to discontinuous change in the slope of the contact force for $g_n>0$. For certain numerical simulations, the discontinuity in slope and the sudden change in the magnitude of contact force might lead to additional iterations or possible divergence issues. Hence, a quadratically regularised penalty law is often used to ensure a smooth transition in the contact forces as given by,

32 $$\begin{aligned} n_c(g_n) = {\left\{ \begin{array}{ll} \bar{n}- p_{n} \ g_{n}, &{} g_{n} \le 0 \\ \frac{p_n}{2 \bar{g}} g_n^2 - p_ng_n + \bar{n}, &{} 0 < g_n \le \bar{g}, \quad \bar{n} = \frac{p_n \bar{g}}{2}\\ 0, &{} g_{n} > 0 \end{array}\right. } \end{aligned}$$

In Eqn. 32, $\bar{g}$ is a positive gap whose value is in the range $\bar{g}\approx 0.1R$, where *R* is the average radius of the two beams in contact.

#### Augmented Lagrangian method

In the penalty method for contact formulation, achieving a penetration value $g_n$ of 1-5% of the beam radius is desirable. Ideally, as $p_n \rightarrow \infty$, $g_n$ should approach zero. However, a numerically large penalty stiffness value may lead to matrix system ill-conditioning, causing numerical divergence. Estimating a single penalty stiffness value for an entire simulation, ensuring acceptable penetration after each load step, is challenging, especially for complex contact scenarios. A rough estimate of the length-specific apparent penalty stiffness $p_n$ can be derived using the Hertz contact theory for cylinders [8] as

33 $$\begin{aligned} p_n = \frac{\pi E}{8(1-\nu ^2)} \end{aligned}$$

In certain contact scenarios, the estimated penalty stiffness value may not guarantee convergence, requiring the user to resort to a ‘trial-and-error’ method for finding the optimum penalty stiffness. An alternative is using the Lagrange Multiplier method or augmented Lagrangian contact constraining techniques. While the Lagrange multiplier method satisfies KKT conditions exactly (Eq. 13), estimating additional Lagrange multipliers increases computational expense. Researchers [43, 44] developed the augmented-Lagrangian contact constraint method, combining penalty method and Lagrange multiplier advantages to achieve a near-zero penetration for a finite $p_n$. The equation for contact force magnitude $n_c(g_n)$ in the augmented-Lagrangian approach during a $k-$th Newton–Raphson iteration is given by,

34 $$\begin{aligned} n^{k}_c(g_n) = {\left\{ \begin{array}{ll} -\lambda ^{k} + p_n g_n^{k}, &{} g_{n}^{k} \le \frac{\lambda ^k}{p_n} \\ 0, &{} g_{n}^{k} > \frac{\lambda ^k}{p_n} \end{array}\right. } \end{aligned}$$

where $\lambda ^k$ is a known Lagrange multiplier for the $k-$th iteration that is updated using the equation (at time $t=0$, $\lambda = 0$),

35 $$\begin{aligned} \lambda ^{k} = \lambda ^{k-1} - \min \left( p_n g_n^{k}, \lambda ^{k-1} \right) \ \forall \ k > 1 \end{aligned}$$

In Eq. 35, $\lambda ^{k}$ is updated using the $k-$th iteration of gap function $g_n$ instead of the $(k-1)$-th iteration value. In order to allow stabilisation of the contact force ($n_c$) prediction and to avoid convergence issues, the value of $\lambda ^{k}$ is updated after the first two Newton–Raphson iterations. The value of $\lambda$ at the start of a new time step is set to the converged value of $\lambda$ from the previous time step. Therefore, using the Eqs. 34 and 35, the contact force acting on interface of the beam bodies can be manipulated without changing the specified penalty stiffness ($p_n$) value.

##### Remark

In the current work, the Lagrange multiplier $\lambda ^{k}$ is *simultaneously* updated within the Newton–Raphson loop. This technique of updation also known as the Uzawa iteration scheme [44, 45], affects the quadratic convergence of the Newton–Raphson method. An alternative to preserve the Newton–Raphson convergence is to use a nested algorithm as presented in Table 1 of Simo et al. [44], where $\lambda ^{k}$ is separately updated in an outer loop within which the Newton–Raphson loop is solved.

## Numerical solution procedure

The final form of the discretised equilibrium equations (Eq. 9) can be rearranged after substituting the linearised expressions for spatial internal forces and moments (Appendix A) and rearranging the terms for a computational node $\mathrm C$. The linearised contributions of the contact force $\varvec{n}_c$ either from the point-to-point (Eq. 19) or line-to-line (Eq. 29) beam contact and the contact moment $\varvec{m}_c$ also have to be added for the CVs that are identified to be in contact using the contact search algorithm. Therefore, the final form of a system of equilibrium conditions read as follows,

36 $$\begin{aligned} \varvec{A}_\textrm{W} \begin{bmatrix} (\Delta \varvec{w})_\textrm{W}\\ (\Delta \varvec{\psi })_\textrm{W} \end{bmatrix} + \varvec{A}_\textrm{C} \begin{bmatrix} (\Delta \varvec{w})_\textrm{C}\\ (\Delta \varvec{\psi })_\textrm{C} \end{bmatrix} + \varvec{A}_\textrm{E} \begin{bmatrix} (\Delta \varvec{w})_\textrm{E}\\ (\Delta \varvec{\psi })_\textrm{E} \end{bmatrix} = \begin{bmatrix} (\varvec{R}^{\varvec{w}})_\textrm{C}\\ (\varvec{R}^{\varvec{\psi }})_\textrm{C} \end{bmatrix} \end{aligned}$$

where $\varvec{A}_\textrm{C}$ is a coefficient matrix containing the contributions of node $\mathrm C$ while the matrices $\varvec{A}_\textrm{W}$ and $\varvec{A}_\textrm{E}$ represent the interactions of cell $\text {C}$ with the neighbouring cell centres $\mathrm W$ and $\mathrm E$. The right-hand side of the Eq. 36 is the source vector contribution. The source vector contains all the explicit contributions of a computational cell $\text {C}$. All the coefficient matrices are $(6 \times 6)$ dense coupled matrices with the primary unknowns being $\Delta \varvec{w}$ and $\Delta \varvec{\psi }$. The three components of the mean line displacement correction and cross-section rotation vectors have to be solved in a coupled manner. The details of the diagonal and off-diagonal coefficient matrices coming from the internal forces and moments are provided in the Appendix section of Bali et al. [16] paper.

The linearised Eqs. 36 are assembled for all CVs forming a system of equations given by,

37 $$\begin{aligned} \big [ \varvec{A} \big ] \big [\varvec{\phi }\big ] = \big [\varvec{R}\big ] \end{aligned}$$

resulting in $6M \times 6M$ sparse matrix $[\varvec{A}]$ with weak diagonal dominance, where *M* is the total number of CVs. The coefficients $\varvec{A}_\textrm{C}$ constitute the diagonal of $[\varvec{A}]$ whereas matrices $\varvec{A}_\textrm{W}$ and $\varvec{A}_\textrm{E}$ contribute to its off-diagonal terms. The solution vector $\big [\varvec{\phi }\big ]$ contains the primary unknowns $\Delta \varvec{w}$ and $\Delta \varvec{\psi }$, and $\big [\varvec{R}\big ]$ is the source vector containing the explicit discretised terms and boundary condition contributions. The final system of linearised algebraic equations, obtained by assembling Eq. 36 for all control volumes in the mesh, is solved using a C++ based Eigen library [46].

For every pseudo-time increment, the coupled equations are iteratively solved by Newton–Raphson procedure, until a user-defined convergence tolerance is achieved within the restriction of user-specified maximum number of allowable iterations. For convergence, both the Euclidean norms of the solution increment vectors ($\Vert \varvec{\phi }\Vert$) and residuals from the linear system of equations ($\Vert \varvec{R}\Vert$) are checked. Since the displacements are additively updated and the rotations in a multiplicative manner, the norms of displacement correction vector ($\Vert \Delta \varvec{w} \Vert$) and the rotation correction vector $\Vert \Delta \varvec{\varvec{\psi }} \Vert$ are separately calculated and the solution increment residual is set as $\Vert \varvec{\phi }\Vert = \max (\Vert \Delta \varvec{w} \Vert ,\Vert \Delta \varvec{\varvec{\psi }} \Vert )$. For convergence of the solver, after each iteration, either of the two norms have to fall below a prescribed tolerance, i.e. $\Vert \varvec{R}\Vert < \delta _{\varvec{R}}$ and $\Vert \varvec{\phi }\Vert < \delta _{\varvec{\phi }}$. The current method has been implemented in open-source software OpenFOAM ([47]) (version foam-extend-4.1), exploiting the developed object oriented FV procedures.

The overall solution procedure can be summarised as follows,

1. Initialise mean line position vector $\varvec{r}$ and rotation tensor $\varvec{\Lambda }_t$ using Eq. 3 and 4 values from the previous time-step or initial conditions for the first time-step.
2. Enter the current (pseudo-) time-step $t_n$ loop.
3. Enter the Newton–Raphson iteration loop.
  - Calculate the explicit values (evaluated from previous Newton-Raphson iteration) and the coefficients of implicit contributions for internal forces and moments in all the internal beam cells.
  - Check for contact; if more than one beam is found, render contact active and proceed with the contact search.
    (i) Create C^1^-continuous Hermite splines for contact detection.
    (ii) Update the list of cell pairs for two neighbouring beams in contact using the point-to-point or line-to-line contact search procedure as discussed in Appendix C.
    (iii) Evaluate the contributions of the linearised contact force (Eq. 19, 29) as well as contact moments that need to be added to the system of equations for the searched contact pair.
  - Update the boundary conditions (see Section 3.3 of Bali et al. [16]).
  - Assemble the system of equations (Eq 37), add contact contributions at the appropriate cell locations and solve the system using the Eigen direct solver.
  - Update beam kinematics, strain measures, and stress-resultants (see Algorithm 2 in Bali et al. [16]).
  - Check convergence criteria and return to step (3) if predefined tolerance is not achieved.
4. Proceed to the next time-step $t_{n+1}= t_n + \Delta t$ (return to step 1) if final time-step is not reached.

## Numerical verification cases

This section investigates several numerical test cases to establish the solver’s ability to handle point-to-point and line-to-line contact between beams. Section 6.1 explores various aspects of point contact involving (a) sliding, (b) occurrence of multiple contacts, (c) mesh-dependence studies, and (d) convergence of the Newton–Raphson solver. Section 6.2 investigates all the above-mentioned aspects when the beams are almost parallel in configuration. Numerical test cases adopted from the literature are used to verify the finite volume numerical contact solver, and comments regarding the performance and accuracy of the solver are presented.

For comparing the numerically obtained results with the reference, a percentage relative error is calculated as,

38 $$\begin{aligned} \text {\% relative error} = \bigg | \frac{\tau ^{\text {num}} - \tau ^{\text {ref}}}{\tau ^{\text {ref}}} \bigg | \times 100\% \end{aligned}$$

For comparison purposes, numerical results of several orders of magnitude fine mesh are adopted as reference. All the test cases have been executed using a quad-core CPU with hyper-threading (Intel Core(TM) i7-8565U CPU with base frequency 1.80GHz and maximum turbo frequency 4.6GHz).

**Fig. 6:**
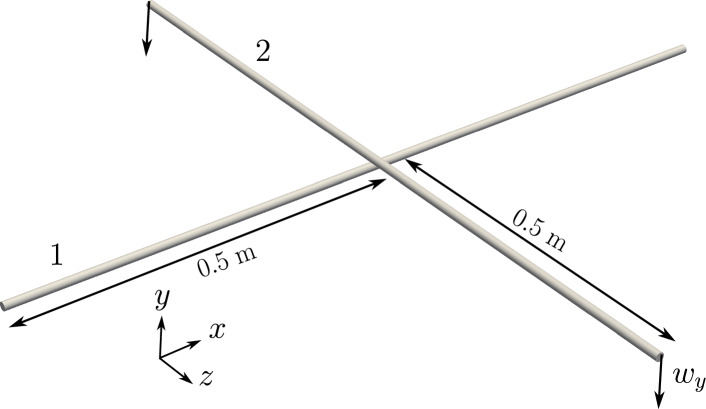
Two perpendicular beams in contact: initial configuration

**Table 1:** Two perpendicular beams in contact: geometric properties

	Cross-section (cm)	*A* (cm^2^)	*I* (cm^4^)	Scaling factor
Square [18]	Side, $a=1$	1	$8.333 \times 10^{-2}$	–
Equivalent circle	Radius, $R=0.56418958$	1	$7.957747 \times 10^{-2}$	1.047195

### Point-to-point contact examples

The following numerical test cases investigate the performance and robustness of the point-to-point beam contact formulation adapted for the finite volume framework.

1. Two perpendicular beams in contact at a single point: a no sliding point-contact benchmark test case is presented where mesh convergence of the contact force is studied. Both linear penalty and augmented Lagrangian contact constraint methods are investigated for this test case. Additionally, using this case, it is demonstrated that, in the limit of linear analysis, the predicted nonlinear contact force converges to the analytical solution available for the linear regime.
2. Two cantilever beams placed perpendicular to each other are subjected to sliding contact: this test case demonstrates the ability of the solver to handle frictionless sliding, where loads and reaction forces developed in the beams become unsymmetrical due to sliding.
3. Building a $5 \times 5$ net configuration: This example is a more-involved contact problem where the capabilities of the numerical contact solver to handle multiple point contacts during the deformation of beams are investigated.

#### Example 1: two perpendicular beams in contact at a single point

This first numerical example is adapted from Zavarise et al. [18], where the test case is investigated for both frictionless and frictional point contact. Here, the results for frictionless contact are presented. Two orthogonal steel beams of equal lengths $L_1 =L_2 = L=1$ m and material properties, $E=210$ GPa and $\nu =0.3$ are placed perpendicular to each other in their initial undeformed state as shown in Fig. 6. The beam 1 in its initial configuration touches the beam 2.

In Zavarise et al. [18], the geometric properties of the beam, like area (*A*) and second moment of area (*I*), are calculated assuming a square cross-section of side-length 1 cm. However, a circular cross-section with a radius $R=10$ cm was used to visualise the deformed beam geometry. To be consistent with the test case and to avoid choosing two different cross-sections for geometry and visualisation, beams are assumed to be circular with equal radii; the radius is calculated from the area of a square cross-section of side length 1 cm and a scaling factor is used for the second moment of area so that the value is equivalent to the square cross-section (Table 1).

**Fig. 7:**
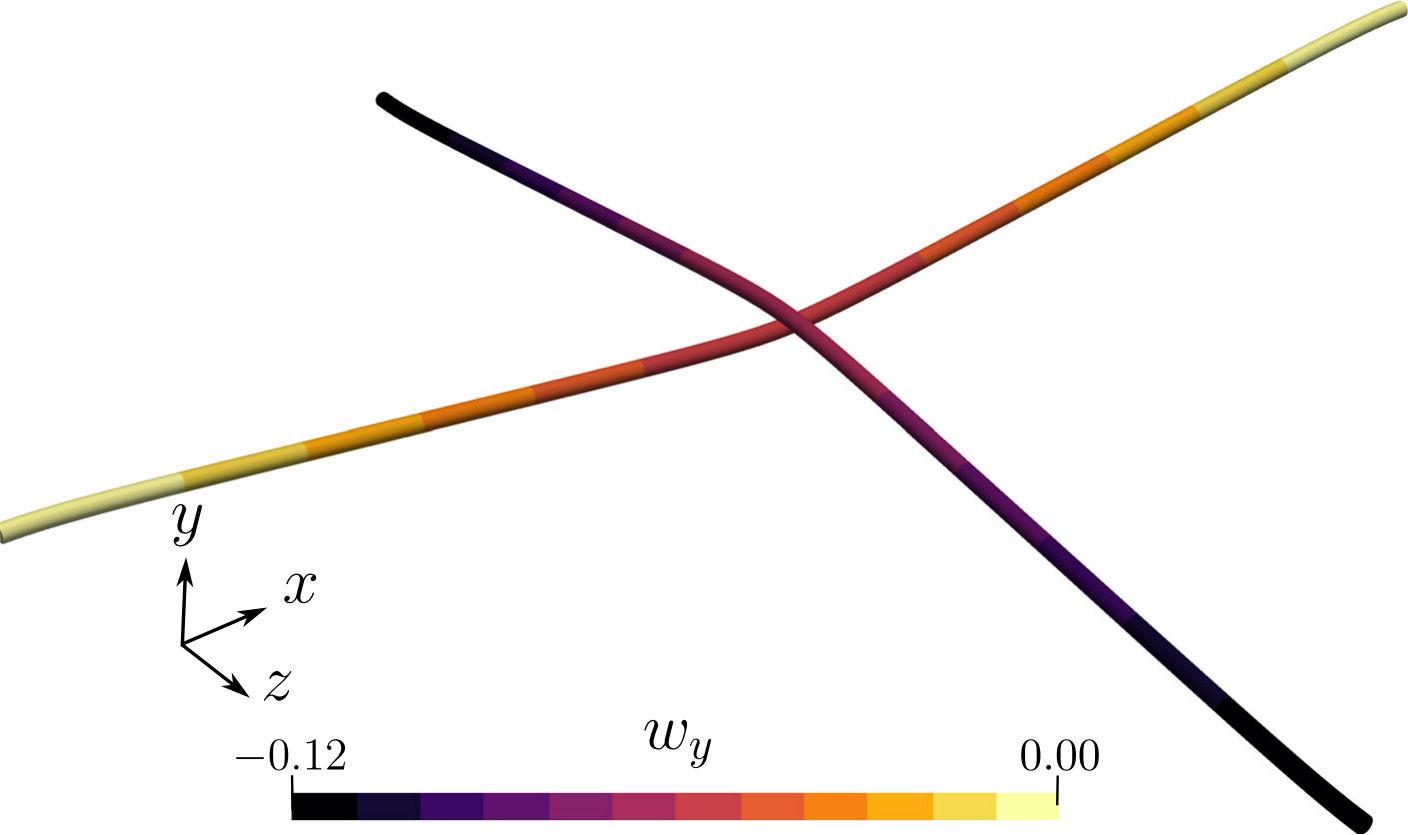
Two perpendicular beams in contact: final deformed configuration at the end of $t=6$ s

Both ends of the beam 1 (Fig. 6) are clamped; the beam 2 is subjected a displacement in the y-direction, $w_y = -0.12$ m. The rotation of the cross-section for the beam 2 at both ends is restricted. A linear penalty law is adopted for the contact algorithm with the stiffness value, $p_n=1 \times 10^8$ N/m. The simulation is run for a total time, $t=6$ s, and the $y-$displacement on beam 1 is applied in 60 increments. The deformed configuration of the beams at the end of the time step is shown in Fig. 7.

**Fig. 8:**
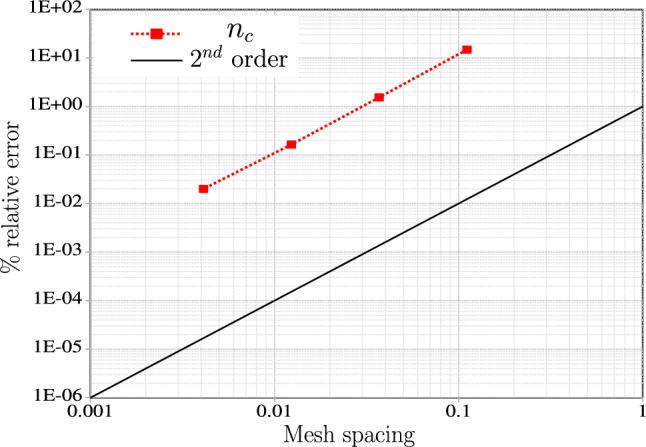
Two perpendicular beams in contact: mesh convergence plot of contact force magnitude for an applied end displacement $w_y=-\,0.12$ m

The test case is repeated for successive mesh refinements in the order of $3^{\textrm{n}}$, i.e., $9, \ 27, \ 81, \ 243$ CVs; the variation of contact force magnitude and the penetration value for increasing mesh density is provided in Table 2. An average of $4-6$ Newton-Raphson iterations for 27 CVs are required for the solution increment vector norm ($\Vert \varvec{\phi } \Vert$) to converge below $1 \times 10^{-10}$; the simulation execution time is less than 7 s. Table 3 shows the force residual norm ($\Vert \varvec{R} \Vert$) at two different (pseudo-) times. Figure 8 shows the contact force’s relative % mesh error due to the successive reduction in mesh sizes; a quadratic force convergence is observed. For calculating the % mesh error, a refined mesh of $3^{7}$ CVs is used as reference values.

**Fig. 9:**
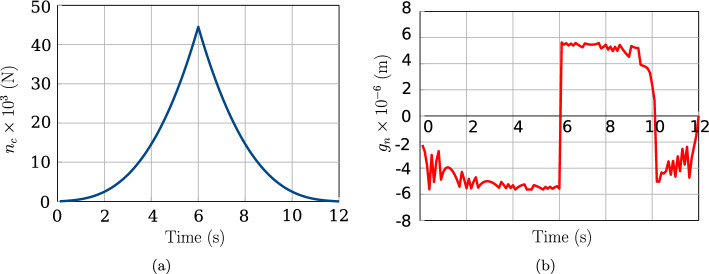
Two perpendicular beams in contact: variation of contact force (left) and penetration (right) values using the augmented Lagrangian approach

The test case is repeated by activating the augmented Lagrangian contact algorithm method instead of the linear penalty constraint. Additionally, after $t=6$ s, beam 2 is unloaded by reducing the applied $y-$displacement at the same rate for the next 6 s. The reason behind setting up the unloading stage is to demonstrate the effectiveness of the augmented Lagrangian method for the full range of motion. So, the expected result at the end of $t=12$ s is the beams returning to their initial configuration with zero contact force. The penalty stiffness value is reduced to $p_n=5\times 10^4$ N/m. For a conventional penalty method, this low penalty value leads to ‘passing through’ of the two beams after a time, $t=1.5$ s. However, upon activating the augmented Lagrangian method, the entire simulation runs smoothly for both the loading and unloading stages of the beam deformation. The variation of contact force magnitude during the simulation for activated augmented Lagrangian is shown in Fig. 9a for 27 beam CVs, and the corresponding penetration values are presented in Fig. 9b.

**Table 2:** Two perpendicular beams in contact: variation of the contact force magnitude and penetration ($g_n$) at the last load step for increasing mesh density ($p_n = 1 \times 10^8$ N/m)

CV	$n_c$ (N)	$g_n$ (m)
9	49836.4	$-4.983 \times 10^{-4}$
27	44072.8	$-4.407 \times 10^{-4}$
81	43475.9	$-4.348 \times 10^{-4}$
243	43413.3	$-4.341 \times 10^{-4}$

**Table 3:** Two perpendicular beams in contact: residual force norm $\Vert \varvec{R} \Vert$ for two stages of loading (27 CVs)

$t = 1.5$ s		$t = 6$ s	
Iterations	$\Vert \varvec{R} \Vert$	Iterations	$\Vert \varvec{R} \Vert$
0	$1.23 \times 10^{6}$	0	$1.23 \times 10^{6}$
1	$2.09 \times 10^{2}$	1	$2.83 \times 10^{3}$
2	$5.21 \times 10^{1}$	2	$3.71 \times 10^{3}$
3	$1.4 \times 10^{-1}$	3	$7.08 \times 10^{0}$
4	$8.70 \times 10^{-5}$	4	$2.30 \times 10^{-2}$
		5	$7.83 \times 10^{-5}$
		6	$3.16 \times 10^{-7}$

When utilising the augmented Lagrangian constraint, achieving zero penetration up to machine precision may lead to unnecessary iterations. Therefore, a limiting absolute gap function value is set, $g_{\text {lim}} = \pm \ \text {avg} \ (R_1, R_2) \times 10^{-3}$ m. In the current test case ($R_1=R_2 = 0.56$ cm), $g_{\text {lim}} = 5.6 \times 10^{-6}$ m. Within this band of limiting values, the penetration between beams is accepted. Fig 9a shows that absolute penetration values are within the limit of $6 \times 10^{-6}$ m. These values, achieved using the augmented Lagrangian approach with a low stiffness value of $5\times 10^{4}$ N/m, come at the cost of a higher number of Newton-Raphson iterations. At $t=6$ s, the convergence requires 127 iterations, and the execution time is 33.4 s-an expected behaviour for the augmented Lagrangian approach ensuring convergence for lower penalty values but with increased Newton–Raphson iterations.

**Fig. 10:**
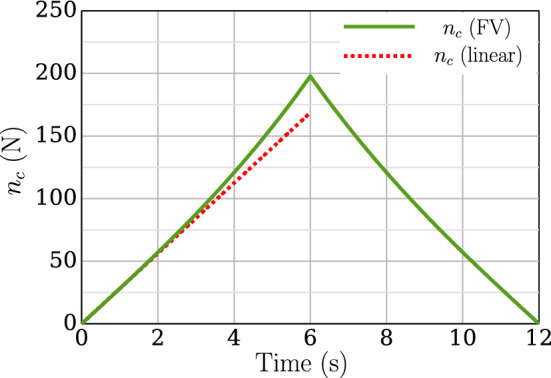
Two perpendicular cantilever beams in contact: nonlinear contact force ($n_c$ (FV)) predicted using the FV point contact formulation for an applied displacement $w_y=-\,0.01$ m at both ends of beam 2. The extrapolated linear prediction ($n_c$ (linear)) is also shown in the graph

The accuracy of the predicted nonlinear contact force using the FV point contact formulation needs to be verified. Since, for the nonlinear regime, the analytical solution for predicting the contact force is unavailable, the following verification study is carried out in the proximity of the linear regime. To achieve a near linear analysis, the *y*-displacement at both ends of beam 2 are reduced to $w_y= -0.01$ m. All other boundary conditions for beams 1 and 2 are the same as mentioned before. A linear penalty law with penalty stiffness $p_n=1\times 10^{8}$ N/m is used. For the applied displacement, the numerical simulation is run for a total time of 12 s, where in the first 6 s, the beam 2 is loaded with displacement $w_y$, and after 6 s, the beam is unloaded till $t=12$ s. The variation of the contact force for the applied displacement is shown in Fig. 10. It is evident from the figure that even for such a small applied displacement, the contact force is still nonlinear. Hence, using the slope of the linear regime, the linear prediction of contact force is extrapolated as shown in the Fig. 10 (red dotted line). The predicted value at $t=6$ s is found to be $n_c \text {(linear)} = 168.6456$ N.

**Fig. 11:**
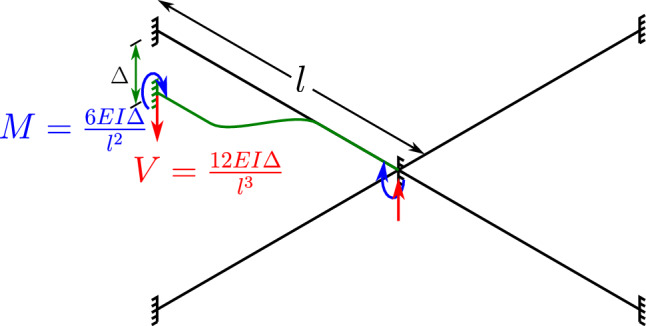
Two perpendicular cantilever beams in contact: schematic for equivalent fixed beam with sinking supports analogy for the numerical test case

The numerical setup of beams for this example are symmetrical. Since both ends of beams 1 and 2 are fixed, and there is no slippage of beam 2 over beam 1, it can be assumed that the point of contact between beams 1 and 2 is also fixed. Now, for the applied end y-displacement on beam 2, this test case is equivalent to a fixed-fixed beam with sinking of supports on one side (schematic in Fig. 11). In the proximity of linear regime, the analytical solution for reaction forces developed due to sinking of support (axial stiffening of beams is ignored here) is given by [48],

39 $$\begin{aligned} V = \frac{12 \ EI}{l^3} \Delta \end{aligned}$$

where *V* is the amount of reaction force generated due to sinking of the support, $\Delta$ is the displacement of the sinking support, *l* is the length of the fixed beam span, and *EI* is the flexural rigidity of the beam. Since the loading and geometric setup of beams 1 and 2 is symmetrical, each beam shares half the applied displacement. Therefore, for the calculation of reaction force for the current numerical simulation, $\Delta = 0.5|w_y| = 0.005$ m, and the length of fixed beam span, $l=0.5 \times$ total length of beam 2, i.e., $l=0.5$ m. The total contact force developed in the mid-location of beam 2 is twice the magnitude of the reaction force *V* because of two fixed beam spans on either side. Ultimately, the contact force prediction via the analytical solution of sinking supports is given by, $(n_c)_p = 2 V$, and plugging in the numerical values, $(n_c)_p$ is found to be 167.9328 N. The percentage error of the numerically extrapolated linear contact force $n_c (\text {linear})$ from Fig. 10 when compared to $(n_c)_p$ is 0.42%. Given the fact that the linear analytical solution ignores axial stiffening due to the sinking supports, the linear extrapolated value of contact force from Fig. 10 is in good agreement with the analytical solution. This numerical study successfully verifies the accuracy of the FV point contact formulation.

#### Example 2: two perpendicular beams in large sliding contact

**Fig. 12:**
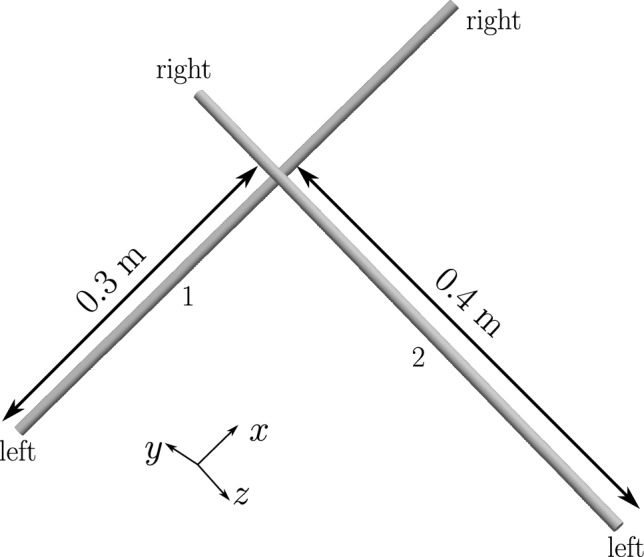
Two perpendicular cantilever beams subjected to sliding: initial configuration

This test case studies the numerical solver’s ability to handle frictionless contact between beams subjected to sliding. Two cantilever beams of circular cross-section with radii $R_1 = R_2=R=0.005$ m, and length $L_1=L_2=L=0.5$ m are considered whose initial undeformed state is shown in Fig. 12.

**Fig. 13:**
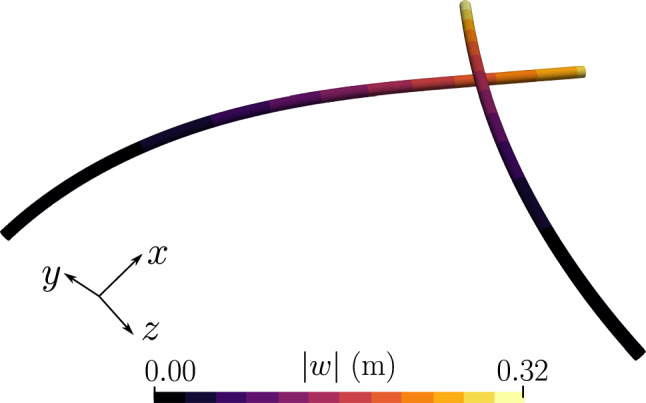
Two perpendicular cantilever beams subjected to sliding: final deformed position of the beams for an applied end load on the right edge of beam 2

The mechanical properties of both the beams are, $E=1\times 10^9$ N/m^2^, and $G=5 \times 10^8$ N/m^2^. The left ends of both the beams are clamped, and at the right end of the beam 2, a concentrated point force, $\varvec{n}\equiv (0,-8,0)$ N is applied in the $y-$direction. A linear penalty law is applied with the penalty stiffness $p_n=1\times 10^5$ N/m, and the total load is applied in 100 increments. Figure 13 shows the final deformed shape of the beams at $t=1$ s; it is evident from the figure that there is significant sliding between the two beams.

It is observed that the Newton–Raphson iterations increase and lose the quadratic convergence with the increase in the amount of sliding per time step between the beams. The possible reason for such behaviour could be the non-smooth nature of the contact, and occurrence of large displacements and rotations due to sliding across multiple beam cells in this specific problem. Additionally, as mentioned before in Sect. 4.4.1, the assumption of constant displacement correction vectors ($\Delta \varvec{w}$ and $\Delta \varvec{\bar{w}}$) across a beam CV also might affect the Newton–Raphson convergence. For an equal mesh discretisation of 20 CVs in both the beams, Table 4 presents the range of iterations for different % loading values for a prescribed convergence tolerance of the solution increment norm, $\Vert \varvec{\phi } \Vert = 1 \times 10^{-7}$. The residual force norm ($\Vert \varvec{R} \Vert$) for two different loading conditions is presented in Table 5.

**Table 4:** Two perpendicular beams subjected to sliding: number of Newton–Raphson (NR) iterations versus the % of total applied for 20 CVs

% Force (%)	NR-iterations
0–22	4–9
23–75	10–15
76–100	16–17

**Table 5:** Two perpendicular beams subjected to sliding: residual force norm $\Vert \varvec{R} \Vert$ for two stages of loading (20 CVs)

20% Force		100% Force	
Iterations	$\Vert \varvec{R} \Vert$	Iterations	$\Vert \varvec{R} \Vert$
0	$8.00 \times 10^{-2}$	0	$8.00 \times 10^{-2}$
1	$3.52 \times 10^{0}$	1	$1.25 \times 10^{0}$
2	$1.21 \times 10^{-1}$	2	$2.65 \times 10^{-1}$
3	$7.14 \times 10^{-3}$	3	$3.06 \times 10^{-2}$
4	$3.33 \times 10^{-4}$	4	$1.42 \times 10^{-2}$
5	$1.74 \times 10^{-4}$	5	$2.48 \times 10^{-3}$
6	$4.36 \times 10^{-6}$	6	$8.37 \times 10^{-4}$
7	$9.05 \times 10^{-6}$	7	$4.80 \times 10^{-4}$
8	$1.36 \times 10^{-7}$	8	$1.05 \times 10^{-4}$
		9	$1.15 \times 10^{-4}$
		10	$2.90 \times 10^{-5}$
		11	$2.82 \times 10^{-5}$
		12	$7.58 \times 10^{-6}$
		13	$6.86 \times 10^{-6}$
		14	$1.88 \times 10^{-6}$
		15	$1.67 \times 10^{-6}$
		16	$4.58 \times 10^{-7}$

**Fig. 14:**
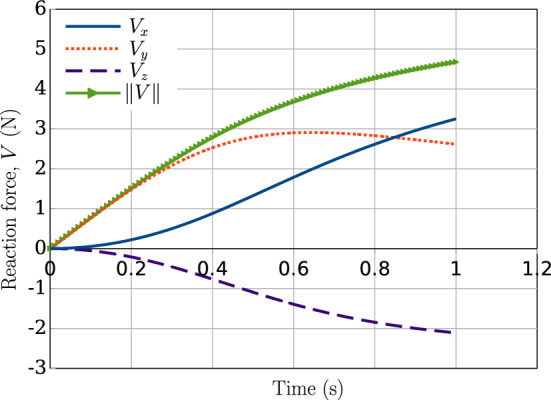
Two perpendicular beams in sliding contact: reaction force (*V*) components and total magnitude generated at the left end of the beam 1

**Fig. 15:**
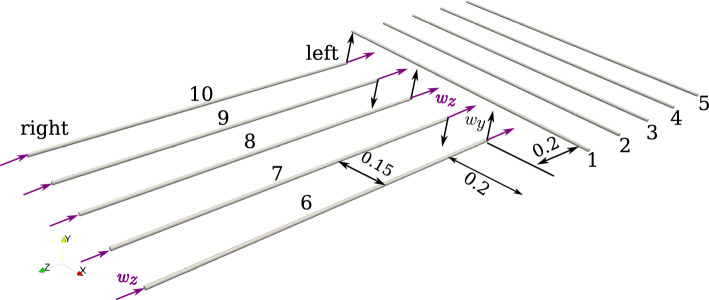
Building a $5\times 5$ net: initial configuration of beams

The simulation execution time for this test case for mesh discretisation of 20 CVs per beam is around 4 s. The norm of the reaction force ($\Vert V \Vert$) along with the individual components at the left end of beam 1 are shown in Fig. 14. The graph for the total magnitude of the reaction force is equivalent to the contact force magnitude developed during the sliding deformation between the beams, i.e., $\Vert V\Vert \equiv \Vert \varvec{n}_c \Vert$. The gradual decrease in the slope of the curve $\Vert V \Vert$ suggests that the beams incur sliding, due to which the contact force does not increase at the same rate as the previous (pseudo) time step.

#### Example 3: building a 5 $\times$ 5 Net

This example investigates the ability of the numerical contact solver to detect multiple points of contact during the simulation, along with its robustness to estimate the contact force(s). The test case is inspired by Zavarise et al. [18], where a similar setup was used for testing frictional point contact. A total of ten beams are taken to set up the initial configuration, as shown in Fig. 15. All the beams have circular cross-sections with the same geometrical and mechanical properties as shown in Table 6.

**Table 6:** Building a $5\times 5$ net: geometrical and mechanical properties of all the beams

Radius (m)	Length (m)	Young’s modulus, *E* (N/m^2^)	Poisson’s ratio, $\nu$
0.005	1	$2.1\times 10^{11}$	0.3

In the initial configuration (Fig. 15), the gap between the beams is set to 0.15 m. The distance between the first beam and the left ends of the perpendicular beams 6–10 is set to 0.2 m. Both ends of beams 1–5 are completely fixed and for beams 6–10, the rotations at both ends are fixed. To build a net, beams 6–10 are subjected to displacements in the global *y* and $z-$direction to simulate a weaving pattern as shown in Table 7.

**Table 7:** Building a $5\times 5$ net: displacement, $w\equiv (w_x,w_y,w_z)$ (m) boundary conditions for the beams $6-10$

Time (s)	Left		Right
	Beams 6, 8, 10	Beams 5, 7	Beams 6–10
$0 < t \le 0.2$	$(0, 0.01, -\,0.2)$	$(0, -\,0.01,-\,0.2)$	$(0, 0, -\,0.2)$
$0.2 < t \le 0.35$	$(0, -\,0.01, -\,0.35)$	$(0, 0.01, -\,0.35)$	$(0, 0, -\,0.35)$
$0.35 < t \le 0.5$	$(0, 0.01, -\,0.5)$	$(0, -\,0.01,-\,0.5)$	$(0, 0, -\,0.5)$
$0.5 < t \le 0.65$	$(0, -\,0.01, -\,0.65)$	$(0, 0.01, -\,0.65)$	$(0, 0, -\,0.65)$
$0.65 < t \le 0.8$	$(0, 0.01, -\,0.8)$	$(0, -\,0.01, -\,0.8)$	$(0, 0, -\,0.8)$
$0.8 < t \le 1$	$(0, -\,0.01, -\,1)$	$(0, 0.01, -\,1)$	$(0, 0, -\,1)$

**Fig. 16:**
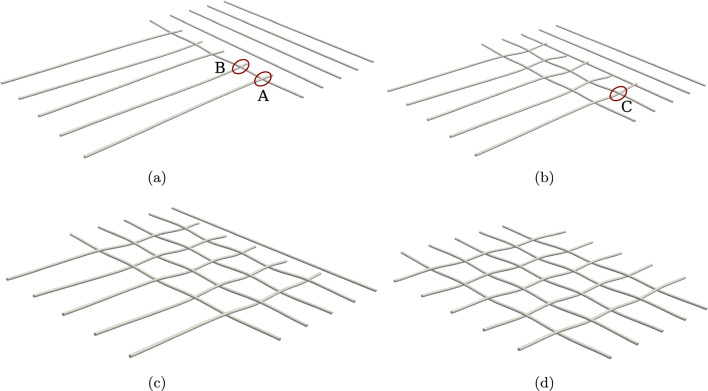
Building a $5 \times 5$ net: deformed configuration of the beams at different time instances, **a** $t=0.25$ s, **b** $t=0.5$ s, **c** $t=0.75$ s, and **d** $t=1$ s

For running the simulation, all the beams are equally discretised into 51 CVs. As mentioned before, the odd number of CVs is to avoid the contact pair detection to lie exactly on the face of the CV leading to numerical divergence. A linear penalty law is used for constraining contact, and the value of penalty stiffness is $p_n=5 \times 10^{7}$ N/m; the (pseudo-)time increment for the simulation is chosen to be $\Delta t = 0.01$ s. Figure 16 shows the deformed configuration of the beams for different time steps, eventually leading to final net formation at $t=1$ s. At time, $t=1$ s, a total of 25 points of contact are identified on the net.

For the prescribed penalty parameter, penetration in the order of $10^{-4}-10^{-5}$ m ($2\%$ of beam radii) is achieved. The average Newton–Raphson iterations for a prescribed solution increment norm $\Vert \phi \Vert = 1 \times 10^{-10}$ from $t=0.21$ s when the contact points are detected is given in Table 8. This solution tolerance value is kept tight to ensure that the residual force norm, $\Vert R \Vert \le 1 \times 10^{-6}$. The total execution time of the numerical simulation for 51 CVs is 155.91 s.

**Table 8:** Building a $5 \times 5$ net: average Newton–Raphson iterations for convergence tolerance, $\Vert \phi \Vert = 1 \times 10^{-10}$ and 51 CVs

Time (s)	Average iterations
$0.21 \le t \le 0.55$	21.8
$0.56 \le t \le 1$	32.3

**Fig. 17:**
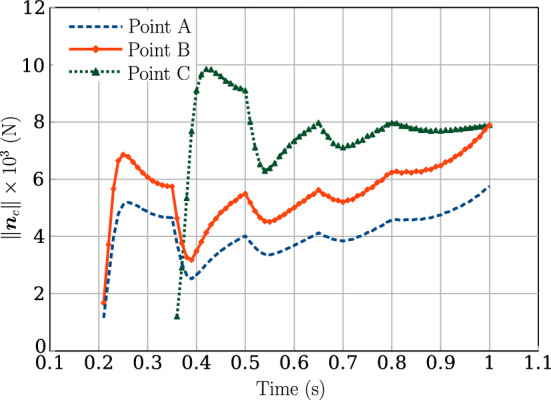
Building a $5 \times 5$ net: variation of the contact force magnitude for contact points A, B and C over time

Figure 17 presents the variation of the magnitude of contact force at three contact points on the beam 1 and beam 2 of the net (highlighted in Fig. 16a, 16b) over the simulation time. The following points can be highlighted from the graph,

- The contact of the perpendicular beams $6-10$ with the beam 1 first occurs at $t=0.21$ s, when the first 5 contact points are detected, and the contact force at both points A and B (Fig. 16a) increases until $t=0.25$ s beyond which the contact force decreases due to sliding between the beams.
- At $t=0.36$ s, contact occurs with the second beam, and the next set of 5 point contacts are identified, after which the contact force at Point C increases. After detecting these points, the contact force at points A and B shows a sudden drop for a few time steps because of additional contact points formed on the net’s second beam.
- This sharp fall in the contact force for Points A, B, and C is seen at consistent times, $t=0.35 \ \textrm{s}, 0.5 \ \textrm{s},0.65 \ \textrm{s}$, and $t=0.8$ s, where in the next time step after these mentioned times, new contact points are detected in the net, thereby resulting in an overall decrease in the contact force for the next few time-steps.

**Fig. 18:**
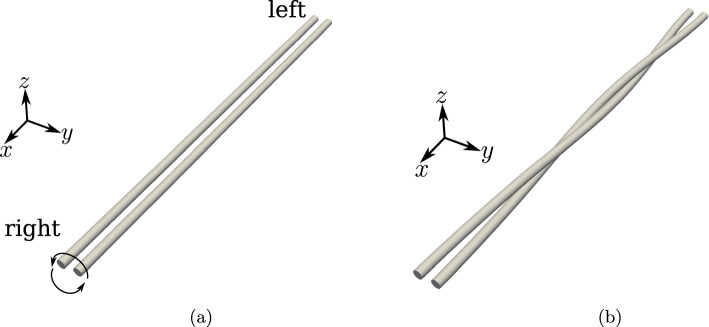
Twisting of two beams: **a** initial configuration of the beams, **b** final deformed beam configuration at the end of $2\pi$ rad twist at the right end of the beams (Length of the beam not in scale)

The numerical test cases when the line contact formalism is prominent are discussed next.

### Line-to-line contact examples

When the contact angle ($\alpha _c$) between the beams is small ( $\alpha _c \lesssim 20^{\circ }$), the unilateral line contact formulation first developed by Meier et al. [21] has to be used. The following numerical test cases are investigated to check the performance and robustness of the line-to-line beam contact formulation adapted for the finite volume framework.

1. Twisting of two parallel beams by $2\pi$ radians: this benchmark numerical example first presented by Meier et al. [21] is used to establish the accuracy of the contact solver to handle possibilities of line contact interaction between beams.
2. Contact interaction between an in-plane 180 degree circular arch and a straight beam: In this example, sliding line contact interaction is studied; the robustness of the solver to handle contact in curved beams and frictionless sliding is investigated.
3. Twisting of two parallel beams by an angle of $1440^{\circ }$ - the last test case presents an extreme twisting deformation of beams where the potential of the contact solver is tested, and the comparison of penetration values and contact forces using penalty method and augmented-Lagrangian method is made.

#### Example 1: twisting of two beams by $2\pi$ rad

Two beams of circular cross-section with radii $R_1 = R_2 = R = 0.01$ m and initial lengths $L_1 = L_2 = L = 5$ m are placed at a separation of $g_0=4R=0.04$ m (the distance is measured from the centroid lines of the two beams) as shown in Fig. 18a.

**Fig. 19:**
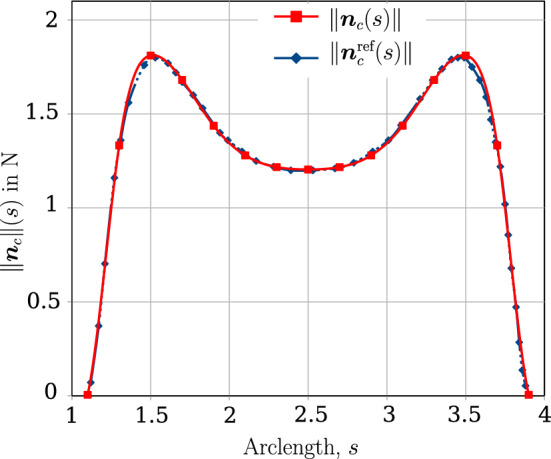
Twisting of two beams: comparison of the contact force magnitude across the beam length for 32 CVs estimated by the contact solver with the reference results in Meier et al. [21]

The mechanical properties of the beam are $E=1 \times 10^9$ N/m^2^ and $G=0.5 \times 10^9$ N/m^2^. The left ends of the beams are completely fixed, whereas at the right end, a Dirichlet displacement boundary condition is applied at the right end wherein the cross-section centres of both the beams circularly rotate about each other until a twist angle of $2 \pi$ rad is achieved. However, the cross-section planes on the right end, are not allowed to rotate. For comparing the contact force distribution with the reference values provided by Meier et al. [21], a regularised penalty law is used with the penalty stiffness value, $p_n = 1000$ N/m^2^ and $\bar{g}=0.1R=0.001$ m. Figure 18b shows the deformed configuration of the beams. Figure 19 compares the contact force distribution across the beam length for a mesh discretisation of 32 beam CVs in both beams; the reference results are adapted from Meier et al. [21] for 32 linear finite element-based beam elements. The figure shows a good agreement of the numerical results with the reference values.

In the Meier et al. [21], the displacements are applied in 8 load steps for a finite element discretisation into 8, 16, 32,  and 64 linear beam elements. However, a minimum number of 5 Gauss integration points are used per element to calculate the gap function and the contact force. So, at least 40 Gauss points are used to establish contact for 8 finite elements. In the finite volume contact solver developed in this thesis, for the line contact formulation, the gap function is evaluated only at the cell centres of the beam CVs, which is equivalent to one Gauss-integration point per element. Hence, the finite volume contact solver generally requires a finer mesh discretisation for convergence. The minimum number of load steps required for convergence for successive mesh refinements for this numerical example is given in Table 9. The table shows that the solver needs more load steps for convergence, even for finer mesh discretisation. The average number of Newton–Raphson iterations when the displacements are applied in 20 load steps is 4.6 per load increment for a prescribed convergence tolerance, $\Vert \phi \Vert = 1\times 10^{-8}$. Table 10 shows the force residual norm ($\Vert \varvec{R} \Vert$) for two different twist angles. The computer simulation time required for an equal mesh discretisation of 64 CVs in both beams is around 1.5 s.

**Table 9:** Twisting of two beams: the minimum number of load steps required for successive mesh refinements to ensure convergence of the numerical simulation

CVs	Load steps
8	40
16	20
32	20
64	20

**Table 10:** Twisting of two beams: residual force norm $\Vert \varvec{R} \Vert$ for two stages of loading (32 CVs)

Twist angle - $\frac{\pi }{2}$		Twist angle - $2\pi$	
Iterations	$\Vert \varvec{R} \Vert$	Iterations	$\Vert \varvec{R} \Vert$
0	$1.78 \times 10^{4}$	0	$1.78 \times 10^{4}$
1	$2.92 \times 10^{-1}$	1	$4.77 \times 10^{0}$
2	$2.35 \times 10^{-2}$	2	$4.69 \times 10^{-1}$
3	$4.09 \times 10^{-7}$	3	$7.24 \times 10^{-4}$
		4	$4.35 \times 10^{-7}$

**Fig. 20:**
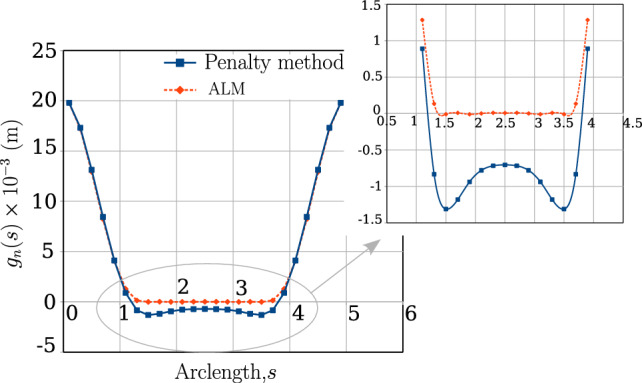
Twisting of two beams: variation of the gap function $g_n(s)$ across the beam length for quadratic penalty and augmented Lagrangian method (ALM). The in-picture graph on the right shows the zoomed region of the gap function variations between length $s=1$ to $s=4$ m

**Fig. 21:**
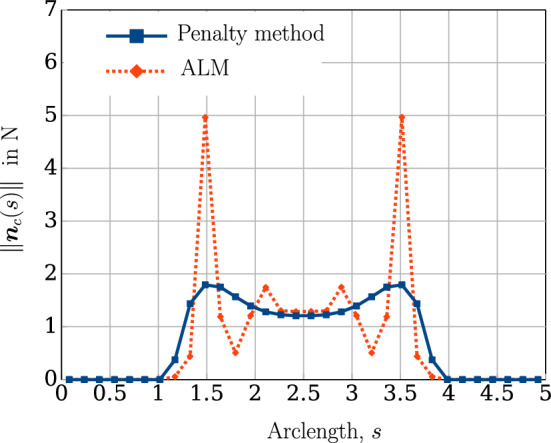
Twisting of two beams: variation of the contact force $\Vert n_c(s) \Vert$ across the beam length for the penalty and augmented Lagrangian (ALM) contact constraint method

Using the penalty parameter $p_n=1000$ N/m^2^ for this test case results in some penetration ($8-15\%$ of beam radius) of the beams. Alternatively, by activating the augmented Lagrangian method (ALM) along with the same penalty stiffness value, the penetration is reduced to the order of $1\times 10^{-6}$ m. Figure 20 presents a comparison of penetration values across the beam length using the penalty method versus the augmented Lagrangian approach. The corresponding comparison of contact force magnitudes is presented in Fig. 21.

Using the penalty law, the maximum penetration occurs at $s=1.5$ m and $s=3.5$ m. For the same penalty stiffness value, when augmented Lagrangian constraint is activated, a spike in the contact force graph at these two beam locations is seen, which results in the reduction of the penetration in the beams. The execution time of the numerical simulation for 64 beam CVs, when using augmented Lagrangian constraint is 6.14 s.

#### Example 2: contact interaction between an in-plane circular arch and a straight beam

**Fig. 22:**
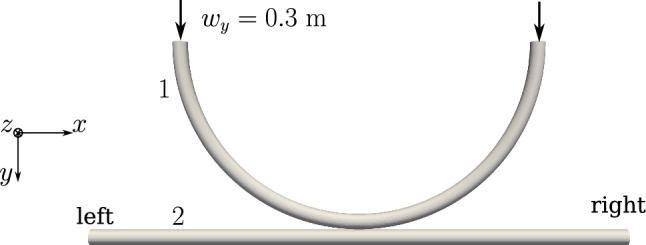
Contact interaction between an in-plane circular arch and a straight beam: initial configuration of beams

The current example was first introduced by Magliulo et al. [31], where the interaction between elliptical beams was studied. Here, the test case is adapted to circular cross-sections of the beams. As shown in Fig. 22, two beams are placed in an initial configuration.

**Table 11:** Contact interaction between an in-plane circular arch and a straight beam: geometrical properties and artificial scaling factors to match the geometric properties of the reference [31]

	Geometry	Area, *A* (m^2^)	Moment of Inertia about *x*, $I_x$ (m^4^)	Scaling factor, $k_x$	Moment of Inertia about *y*, $I_y$ (m^4^)	Scaling factor, $k_y$
Ellipse [31]	Major axis along *x*, $2a = 0.1$ m, Minor axis along *y*, $2b = 0.06$ m	$A = \pi a b = 4.71239 \times 10^{-3}$	$I_x = \frac{\pi }{4} a b^3$ = $1.06029 \times 10^{-6}$	–	$I_y = \frac{\pi }{4} a^3 b = 2.94524 \times 10^{-6}$	–
Circle	Radius, $R=0.03873$ m	$A = \pi R^2 = 4.71239 \times 10^{-3}$	$I_x = \frac{\pi }{4} R^4 = 1.76715 \times 10^{-6}$	0.6	$I_y = \frac{\pi }{4} R^4 = 1.76715 \times 10^{-6}$	1.6666

The length of the straight beam is 2.7 m, and the radius of the circular arch is 0.9 m. The semi-circular arch is placed symmetrically on the straight beam such that it just touches the straight beam in the initial configuration. The major and minor axis of elliptical cross-sections used by Magliulo et al. [31] was 0.1 m and 0.06 m, respectively. Here, for the equivalent elliptical area, the radius for the circular cross-section is found to be $R\approx 0.03873$ m; this value is used for both the arch and the straight beam cross-sections. To use the results from Magliulo et al. [31] for comparison purposes, the second moments of area for the circular cross-section have been scaled according to the elliptical cross-section assumed in the reference. More specifically, Table 11 shows the geometrical properties of the circular cross-section assumed in this example and the corresponding scaling factors used to match the cross-section properties presented in Magliulo et al. [31].

Both the beams have the same mechanical properties, i.e., $E=1\times 10^{11}$ N/m^2^, and Poisson’s ratio, $\nu =0.3$. The rotations are restrained at both ends of the arch and the straight beam; the displacements at the ends of the straight beam are also fixed. A displacement boundary condition is provided at both ends of the circular arch as presented in Table 12.

**Table 12:** Contact interaction of a semi-circular arch and a straight beam: displacement boundary condition applied to both end of the arch

Time (s)	$\varvec{w}\equiv (w_x,w_y,w_z)$ (m)
$$0 \le t \le 1$$	(0, 0.3, 0)
$$1 < t \le 2$$	(0.1, 0.3, 0)

**Fig. 23:**
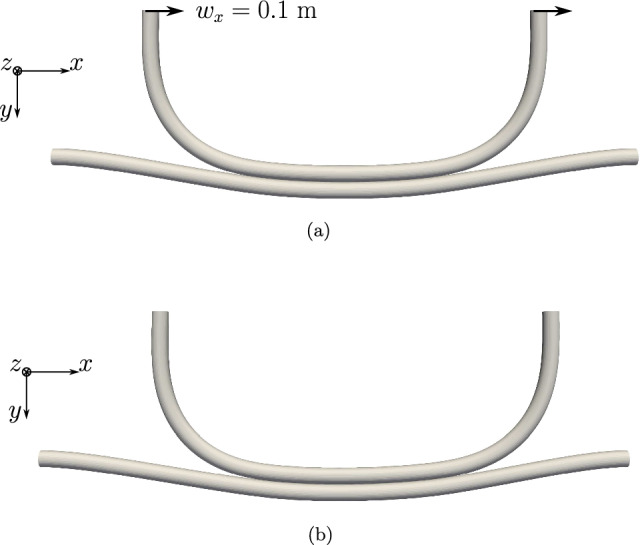
Contact interaction between an in-plane circular arch and a straight beam: **a** deformed configuration at the end of $t=1$ s, and **b** final deformed shape after sliding by 0.1 m along $x-$direction at $t=2$ s

For simulating contact, a quadratic penalty law is used with the stiffness value $p_n=4\times 10^{10}$ N/m^2^ and $\bar{g} = 0.01 R = 0.00038$ m. Three successive mesh discretisation as opted by Magliulo et al. [31] are used here (Table 13) for comparison of the numerical results. The total displacement is applied in 400 increments between time, $0 \le t \le 2$. Figure 23a presents the deformed beam configuration at time $t=1$ s, after which the sliding along $x-$direction starts, and the final deformed shape of the beams at the end of $t=2$ s is shown in Fig. 23b.

**Table 13:** Contact interaction of a semi-circular arch and a straight beam: mesh discretisation values adopted for the test case from the reference [31]

Mesh	Arch	Straight
type	mesh,	beam
	(CVs)	mesh,
		(CVs)
Coarse	90	60
Moderate	120	80
Fine	150	100

**Fig. 24:**
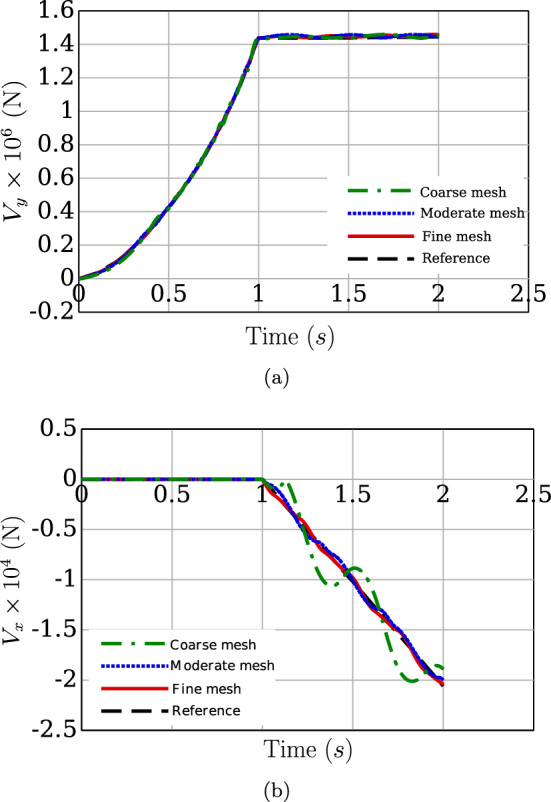
Contact interaction between an in-plane circular arch and a straight beam: **a** sum of the vertical reaction forces ($V_y$) for the two ends of the straight beam along the $y-$direction, and **b** sum of the horizontal reaction forces ($V_x$) for the two ends of the straight beam along the $x-$direction

The deformation of the beams and their contact interaction leads to the development of reaction forces and moments at the fixed ends of both beams. Figure 24a shows the sum of reaction forces in the straight beam along the $y-$direction.

From Fig. 24a, it can be observed that, due to the $y-$displacement of the arch towards the straight beam until $t=1$ s, the reaction forces on the straight beam are observed to increase till $t=1$ s, after which the horizontal sliding starts, and hence, there is no change observed in the $y-$component of the sum of reaction forces at the two ends of the straight beam. Figure 24b presents the sum of horizontal reaction forces developed during the deformation of the beams. During the vertical displacement phase of the arch, the horizontal reaction forces (along $x-$direction) produced in the straight beam are equal and opposite. Hence, their sum is zero until $t=1$ s (Fig. 24b). After that, the horizontal sliding starts, which generates unequal horizontal reaction force components. Therefore, the sum of the horizontal reaction forces is non-zero and an increasing linear trend is observed in Fig. 24b for increasing horizontal sliding. The numerical results from the reference [31] are overlaid in both figures, along with the numerical results for FV contact formulation for three mesh sizes. As evident from Fig. 24b, numerical oscillations in the predicted sum of horizontal reaction forces are present for a lower mesh discretisation, especially during the horizontal sliding stage. These oscillations are drastically reduced for finer mesh sizes. As mentioned previously in Sect. 4.4.2, for the line contact FV formulation, the contact gap is established only from the cell centre of a beam CV, i.e., one Gauss-integration point per CV is used to evaluate the integral line force distributed over the isolated CV length. Therefore, for a lower FV spatial discretisation, numerical errors in the contact force calculations are relatively higher; however, as the mesh is refined, the numerical results of the test case match the reference results reported in the literature.

#### Example 3: twisting of two beams by $8\pi$ rad

**Fig. 25:**
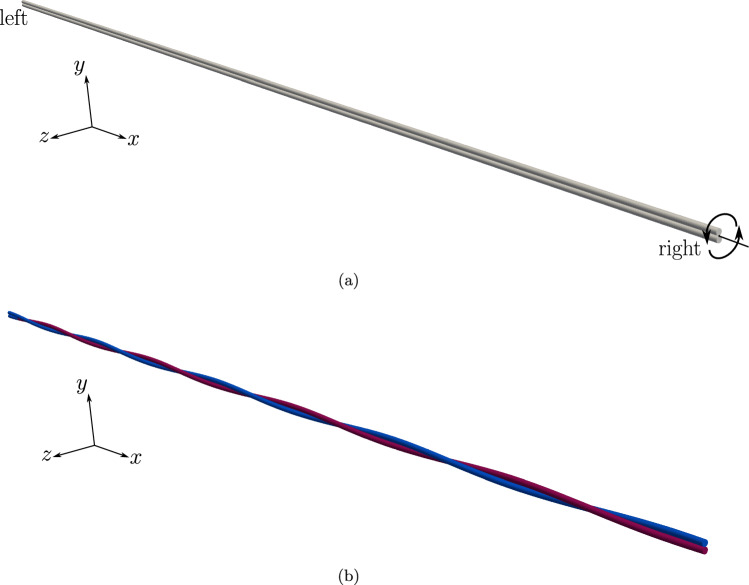
Test case 3—twisting of two beams: **a** initial configuration of the beams, **b** the final deformed configuration at the end of $t=4$ s ($8\pi$ radians)

This example presents an extreme form of twisting between two beams over each other. Other authors have studied similar extreme twist test cases [21–23] to check the robustness of the line contact interaction between beams. Two straight beams of equal radii $R_1=R_2=R=0.01$ m and equal lengths, $L_1=L_2=L=5$ m, separated by an initial gap of 0.0005 m, are taken in the initial configuration (Fig. 25a). The mechanical properties for both beams are $E=1\times 10^9$ N/m^2^ and $G=5\times 10^8$ N/m^2^. The left end of the beam setup is completely clamped (no displacement and rotations are allowed), while at the other end, the beam endpoints are displaced in a circular fashion about the longitudinal beam axis ($x-$axis) up to an angle of $8\pi$ rad keeping the rotational degrees of freedom fixed. The total twist of $8\pi$ rad (four full turns) is applied in 400 increments in a total simulation time of 4 s. For the contact interaction, a quadratic penalty law is used with $p_n=3\times 10^4$ N/m^2^, and $\bar{g}=0.1R=0.001$ m. Figure 25b shows the final deformation for twisting two beams for an extreme deformation of $8\pi$ rad.

This test case is simulated for three different mesh discretisation, i.e., 50, 100,  and 200 CVs. For the applied penalty value $p_n=3\times 10^4$ N/m^2^ and a mesh discretisation of 200 CVs, the maximum penetration at the end of $t=3$ s (three complete turns) is observed to be 0.0016 m (16% penetration into the beam body with respect to the beam radius, $R=0.01$ m). At the end of $t=4$ s, the maximum penetration is around 0.003 to 0.0042 m, amounting to $30-42$ % penetration of beam radius. So, it is evident that the penalty stiffness value provided is not sufficient for such extreme twisting conditions. However, when it is attempted to increase the $p_n$ value beyond $3\times 10^4$ N/m^2^, the numerical simulation does not converge after $t=3.2-3.5$ s.

**Fig. 26:**
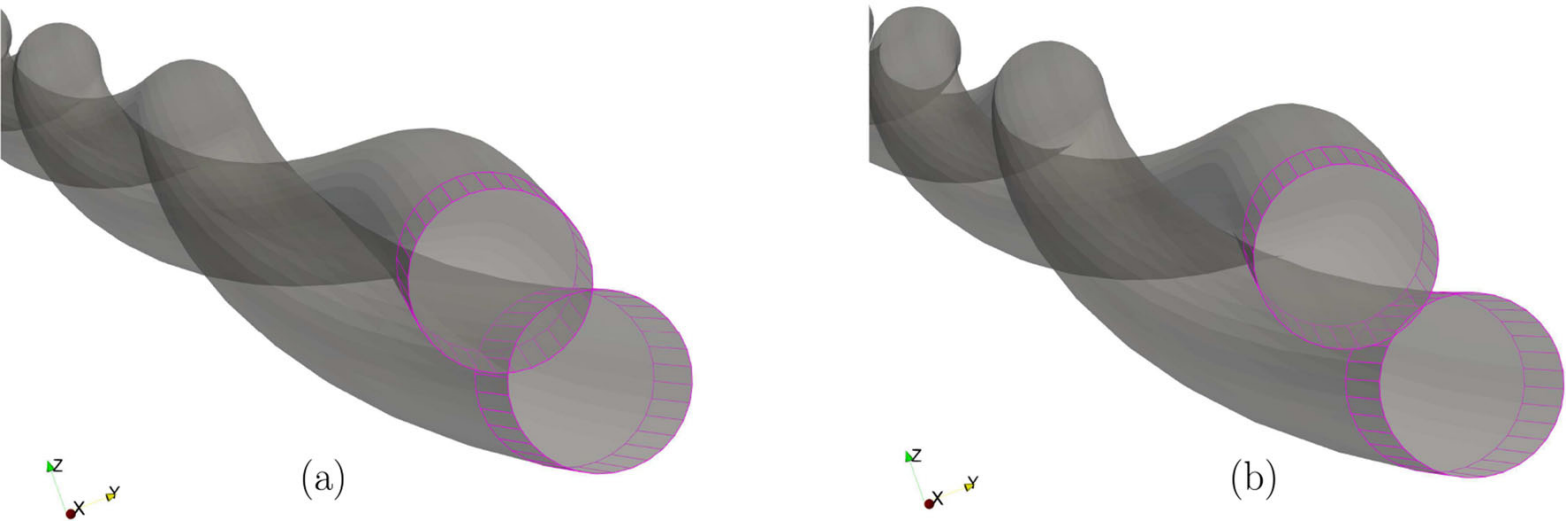
Twisting of two beams by $8\pi$ rad: **a** penetrated cross-section of the beams at $L=2.5$ m when penalty method is used, and **b** the cross-section of the beam setup when the simulation is run by activating the augmented Lagrangian method

Therefore, it was necessary to activate the augmented Lagrangian constraint method to achieve penetration values in the order of $1\times 10^{-6}$ m along the length of the beam. Figure 26 compares the cross-sections of the deformed beams at $L=2.5$ m with and without the augmented Lagrangian constraint.

**Fig. 27:**
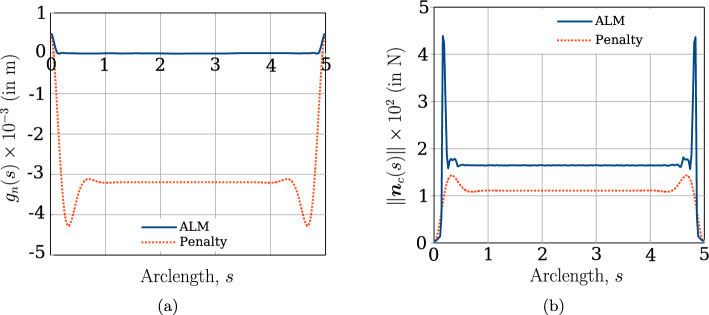
Twisting of two beams by $8\pi$ rad: **a** variation of the penetration value across the beam length for the penalty and ALM constraint, **b** variation of the contact force distribution across the beam length for the penalty and ALM constraint

**Fig. 28:**
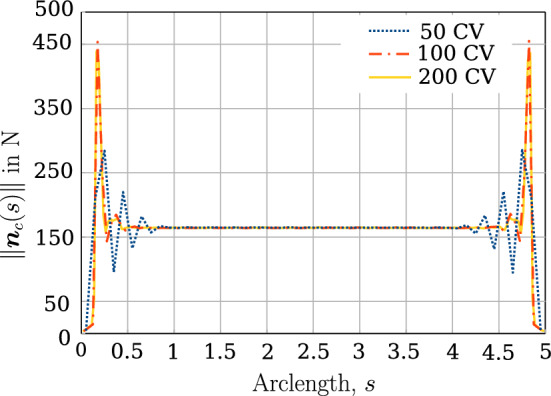
Twisting two beams by $8\pi$ rad: a comparison of contact force magnitude for successive mesh refinements using augmented Lagrangian constraint

The variation of the gap function ($g_n(s)$) and the corresponding contact force magnitude ($\Vert \varvec{n}(s)\Vert$) across the beam length for the penalty and augmented Lagrangian methods are shown in Figs. 27a, 27b respectively. It is evident from Fig. 27b that a higher contact force is estimated for the augmented Lagrangian constraint as compared to the penalty method. The contact force distribution for successive mesh refinements of 50, 100, 200 CVs for the augmented Lagrangian contact algorithm is presented in Fig. 28. Some oscillations in the contact forces near the beam endpoints are observed for coarser mesh discretisation. Due to the presence of higher values of the gap function in that region, the contact solver estimates a large contact force which, due to the coarser mesh, shows an oscillatory pattern that can be reduced by increasing the mesh density, as evident from the Fig. 28. Table 14 presents the average and the maximum number of Newton–Raphson iterations required by augmented Lagrangian contact algorithm for convergence under the prescribed solution norm tolerance, $\Vert \phi \Vert = 1 \times 10^{-8}$. The execution time of the simulation for a mesh of 200 CV is 151.64 s.

**Table 14:** Twisting of two beams by $8\pi$ rad: the average and maximum number of Newton–Raphson iterations for successive mesh refinements using augmented Lagrangian constraint

CVs	Avg. Iterations	Max Iterations
50	7.93	19
100	9.12	29
200	10.04	28

## Conclusion

This article introduces a novel finite volume approach for simulating mechanical contact between beams, employing a geometrically exact nonlinear Simo-Reissner mathematical model. Point-to-point and line-to-line contact formulations are utilised, with nonlinear contact forces linearized and solved implicitly using a Newton–Raphson iterative scheme. Three point-to-point and three line-to-line benchmark test cases are simulated to validate the proposed method, covering scenarios such as sliding, twisting, and multiple contacts. Numerical results are compared with standard finite element solutions, yielding insights into the method’s performance. The following observations are made from the numerical analyses:

- The proposed finite volume (FV) beam contact procedure demonstrates accurate and robust predictions for both point and line contact scenarios, with efficiency influenced by spatial discretisation. A reasonable number of Newton–Raphson iterations are sufficient to attain a good convergence of the results.
- Second-order accuracy in contact force prediction is observed for successive mesh refinement in the standard two perpendicular beams contact test case (Example 6.1.1).
- In line-to-line beam contact, higher mesh discretisation is typically required for comparable results to conventional finite element methods due to the evaluation of a single gap function at the cell centre of the discretised beam.
- In challenging contact scenarios, where the choice of penalty stiffness is not straightforward and the penalty method may result in undesirable penetration (Example 6.2.3), the augmented Lagrangian contact constraint method is preferred for better contact force distribution and can also be used as an alternative to achieve low penetration with a reasonably low penalty stiffness parameter (Fig 9a in Example 6.1.1).

The finite volume discretisation of beam-to-beam contact for frictionless cases presented in the current work shows the efficacy of the finite volume methods in addressing mechanical contact interactions in flexible slender structures. These FV beam contact procedures integrated with the existing FV-based geometrically exact beam solver (Bali et al. [16]) developed in an open-source software OpenFOAM [47] open new pathways to solve complex fluid–structure simulations such as modelling fish-net interactions with oceanic waves and slender plant growth in marine environments within a single finite volume framework for both solid and fluid domains. Extensions of the current formulation to frictional contact will be addressed in the future.

## Figures and Tables

**Fig. 29:**
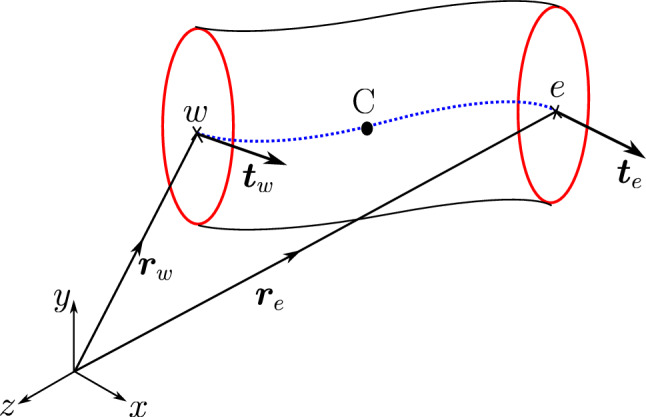
Hermite spline representation for an isolated control volume of a beam

**Fig. 30:**
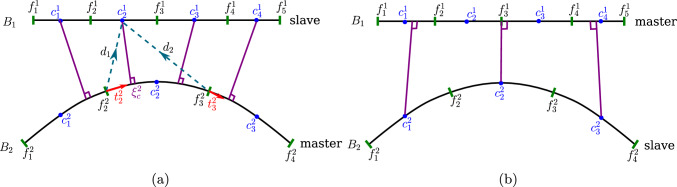
Line-contact search procedure between a pair of beams: **a** first half-pass, **b** second half-pass

## Linearisation of stress-resultants

To linearise the spatial forces $\varvec{n}$ and moments $\varvec{m}$, a systematic use of the directional derivatives for the kinematic quantities ($\varvec{r}, \varvec{\Lambda }_t$) and the strain measures ($\varvec{\Gamma }, \varvec{K}$) is necessary. For a given beam configuration, the linearisation of perturbed kinematic quantities $\big (\varvec{r}_\epsilon = \varvec{r}_0 + \varvec{w} + \epsilon \Delta \varvec{w},(\varvec{\Lambda }_t)_\epsilon = \exp (\epsilon \widehat{\Delta \varvec{\psi }}) \varvec{\Lambda }_t\big )$ yields,

A1 $$\begin{aligned} \frac{d}{d\epsilon } \Big (\varvec{r}_\epsilon \Big ) \bigg |_{\epsilon = 0} = \Delta \varvec{w} \qquad \qquad \frac{d}{d\epsilon } \Big ((\varvec{\Lambda }_t)_\epsilon \Big ) \bigg |_{\epsilon = 0} = \widehat{ \Delta \varvec{\psi }} \ \varvec{\Lambda }_t\end{aligned}$$

where $\epsilon$ is a small scalar perturbation. The linearised forms for the perturbed (material) strain measures $\varvec{\Gamma }_\epsilon = (\varvec{\Lambda }_t^\textrm{T})_\epsilon \varvec{r}^\prime _\epsilon - \varvec{e}_1$ and $\varvec{K}_\epsilon = (\varvec{\Lambda }_t^\textrm{T})_\epsilon (\varvec{\Lambda }_t^\prime )_\epsilon$ follows from Simo et al. [39] as,

A2 $$\begin{aligned}{} & {} \frac{d}{d\epsilon } \Big (\varvec{\Gamma }_\epsilon \Big ) \bigg |_{\epsilon = 0} =(\varvec{\Lambda }_t)^\textrm{T} \widehat{(\varvec{r})^\prime } (\Delta \varvec{\psi }) +(\varvec{\Lambda }_t)^\textrm{T} \Delta \varvec{w}^\prime \end{aligned}$$

A3 $$\begin{aligned}{} & {} \frac{d}{d\epsilon } \Big ( \varvec{K}_\epsilon \Big ) \bigg |_{\epsilon = 0} =(\varvec{\Lambda }_t)^\textrm{T} \Delta \varvec{\psi }^\prime \end{aligned}$$

Using these linearised strain measures, the spatial force, $\varvec{n} = \varvec{\Lambda }_t\varvec{C}_\textrm{N} \varvec{\Gamma }$, upon linearisation yields,

A4a $$\begin{aligned}&\textrm{L}[\varvec{n}] = \overset{*}{\varvec{\Lambda }}_t\varvec{C}_\textrm{N} \overset{*}{\varvec{\Gamma }} + \frac{d}{d\epsilon } \Big ( (\varvec{\Lambda }_t)_\epsilon \varvec{C}_\textrm{N} \varvec{\Gamma }_\epsilon \Big ) \bigg |_{\epsilon = 0} \end{aligned}$$

where $\overset{*}{(\cdot )}$ denotes the *known* fields from the previously converged Newton-Raphson iteration. The first term in the RHS of the above equation is the explicit (*unbalanced*) force evaluated using the previous converged values of kinematic quantities and strains; the second part of the equation is the directional derivative of force, which on further expansion leads to,

A4b $$\begin{aligned}&\implies \textrm{L}[\varvec{n}] = \overset{*}{\varvec{\Lambda }}_t\varvec{C}_\textrm{N} \overset{*}{\varvec{\Gamma }} + \frac{d}{d\epsilon } (\varvec{\Lambda }_t)_{\epsilon } \bigg |_{\epsilon = 0} \varvec{C}_\textrm{N} \varvec{\Gamma } + (\varvec{\Lambda }_t) \varvec{C}_\textrm{N} \frac{d}{d \epsilon } (\varvec{\Gamma }_\epsilon ) \bigg |_{\epsilon =0} \end{aligned}$$

A4c $$\begin{aligned}&\quad = \overset{*}{\varvec{\Lambda }}_t\varvec{C}_\textrm{N} \overset{*}{\varvec{\Gamma }} + \widehat{\Delta \varvec{\psi }} \ \left( \varvec{\Lambda }_t\varvec{C}_\textrm{N} \varvec{\Gamma } \right) + \left( \varvec{\Lambda }_t\varvec{C}_\textrm{N}(\varvec{\Lambda }_t)^\textrm{T} \right) \nonumber \\&\qquad \Big [ \Delta \varvec{w}^\prime + \widehat{(\varvec{r})^\prime } \ (\Delta \varvec{\psi }) \Big ] \end{aligned}$$

The coefficient of $\widehat{\Delta \varvec{\psi }}$ in Eq. A4c is ‘force’, which is substituted by the evaluated ‘linearised force $\textrm{L}[\varvec{n}]$’ calculated from the previous iteration. For all the other fields in the above equation, known values from previous iteration are used. Hence, the final form of the linearised spatial force is given as,

A4d $$\begin{aligned} \implies \textrm{L}[\varvec{n}]&= \overset{*}{\varvec{\Lambda }}_t\varvec{C}_\textrm{N} \overset{*}{\varvec{\Gamma }} - \widehat{ \textrm{L}[\overset{*}{\varvec{n}}]} \ \Delta \varvec{\psi }+ \big (\overset{*}{\varvec{\Lambda }}_t\varvec{C}_\textrm{N}(\overset{*}{\varvec{\Lambda }}_t)^\textrm{T}\big ) \nonumber \\&\qquad \Big [ \Delta \varvec{w}^\prime + \widehat{(\overset{*}{\varvec{r}})^\prime } \ (\Delta \varvec{\psi }) \Big ] \end{aligned}$$

Similarly, the spatial moment, $\varvec{m} = \varvec{\Lambda }_t\varvec{C}_\textrm{M} \varvec{K}$ can be linearised as,

A5 $$\begin{aligned} \begin{aligned} \textrm{L}[\varvec{m}]&= \overset{*}{\varvec{\Lambda }}_t\varvec{C}_\textrm{M} \overset{*}{\varvec{K}} + \frac{d}{d\epsilon } \Big ( (\varvec{\Lambda }_t)_\epsilon \varvec{C}_\textrm{M} \varvec{K}_\epsilon \Big ) \bigg |_{\epsilon = 0} \\&= \overset{*}{\varvec{\Lambda }}_t\varvec{C}_\textrm{M} \overset{*}{\varvec{K}} + \frac{d}{d\epsilon } (\varvec{\Lambda }_t)_{\epsilon } \bigg |_{\epsilon = 0} \varvec{C}_\textrm{M} \varvec{K} + \varvec{\Lambda }_t\varvec{C}_\textrm{M} \frac{d}{d \epsilon } (\varvec{K}_\epsilon ) \bigg |_{\epsilon =0} \\&\implies \textrm{L}[\varvec{m}] = \overset{*}{\varvec{\Lambda }}_t\varvec{C}_\textrm{M} \overset{*}{\varvec{K}} - \widehat{\textrm{L}[\overset{*}{\varvec{m}}]} \ \Delta \varvec{\psi }+ (\overset{*}{\varvec{\Lambda }}_t\varvec{C}_\textrm{M}(\overset{*}{\varvec{\Lambda }}_t)^\textrm{T}) \ \Delta \varvec{\psi }^\prime \end{aligned}\nonumber \\ \end{aligned}$$

The linearised counterpart of the term $(\varvec{r}^\prime \times \varvec{n})$ in Eq. (2) is given by,

A6 $$\begin{aligned} \begin{aligned}&\textrm{L}[\varvec{r}^\prime \times \varvec{n}] = (\overset{*}{\varvec{r}})^\prime \times (\overset{*}{\varvec{\Lambda }}_t) \varvec{C}_\textrm{N} \overset{*}{\varvec{\Gamma }} + \frac{d}{d\epsilon } \Big ( \varvec{r}^\prime _\epsilon \times (\varvec{\Lambda }_t)_\epsilon \varvec{C}_\textrm{N} \varvec{\Gamma _\epsilon } \Big ) \bigg |_{\epsilon = 0} \\&\qquad \qquad \quad = (\overset{*}{\varvec{r}})^\prime \times (\overset{*}{\varvec{\Lambda }}_t) \varvec{C}_\textrm{N} \overset{*}{\varvec{\Gamma }} + \frac{d}{d \epsilon } (\varvec{r}^\prime _\epsilon ) \bigg |_{\epsilon = 0} \times (\varvec{\Lambda }_t\varvec{C}_\textrm{N} \varvec{\Gamma }) + (\varvec{r})^\prime \\&\qquad \qquad \qquad \times \frac{d}{d \epsilon } \Big ( (\varvec{\Lambda }_t)_\epsilon \Big ) \bigg |_{\epsilon =0} \varvec{C}_\textrm{N} \varvec{\Gamma } + (\varvec{r})^\prime \times \varvec{\Lambda }_t\varvec{C}_\textrm{N} \frac{d}{d \epsilon } \Big ( \varvec{\Gamma }_\epsilon \Big ) \bigg |_{\epsilon =0} \\&\implies \textrm{L}[\varvec{r}^\prime \times \varvec{n}] = \widehat{(\overset{*}{\varvec{r}})^\prime } \ (\overset{*}{\varvec{\Lambda }}_t\varvec{C}_\textrm{N} \overset{*}{\varvec{\Gamma }}) + \biggl [ \widehat{(\overset{*}{\varvec{r}})^\prime } \left( \overset{*}{\varvec{\Lambda }}_t\varvec{C}_\textrm{N}(\overset{*}{\varvec{\Lambda }}_t)^\textrm{T}\right) \\&\qquad \qquad \qquad \qquad \qquad - \widehat{\textrm{L}[\overset{*}{\varvec{n}}]} \biggr ] \Delta \varvec{w}^\prime + \widehat{(\overset{*}{\varvec{r}})^\prime } \biggl [ \overset{*}{\varvec{\Lambda }}_t\varvec{C}_\textrm{N}(\overset{*}{\varvec{\Lambda }}_t)^\textrm{T} \widehat{(\overset{*}{\varvec{r}})^\prime } - \widehat{\textrm{L}[\overset{*}{\varvec{n}}]} \biggr ] \Delta \varvec{\psi }\end{aligned} \end{aligned}$$

The manipulation of skew-symmetric tensors and the corresponding axial vector relation, $\varvec{\theta } \times \varvec{h} = \widehat{\varvec{\theta }}\varvec{h} = -(\varvec{h} \times \varvec{\theta })= - \widehat{\varvec{h}} \varvec{\theta }$ is used in all the above expressions.

## Hermite spline polynomials

The beam mean centreline for an isolated CV is interpolated using cubic Hermite spline polynomials with a local parametric coordinate $\xi$ as follows (Fig. 29),

B1 $$\begin{aligned} \varvec{r}_d(\xi )= & {} H_r^w(\xi ) \ \varvec{r}_w + H_r^e(\xi ) \ \varvec{r}_e + \frac{\Vert \varvec{r}_e - \varvec{r}_w \Vert }{2} \Big [ H_t^w(\xi ) \ \varvec{t}_w \nonumber \\{} & {} + H_t^e(\xi ) \ \varvec{t}_e \Big ] \end{aligned}$$

In Eq. B1, $\varvec{r}_d(\xi )$ represents a discretised centroid line for the CV of the beam, $\varvec{r}_w$ is the position vector of the centre of west face (*w*) and $\varvec{r}_e$ is the position vector of the east face (*e*) centre of the CV. $\varvec{t}_w$ and $\varvec{t}_e$ is the tangent vectors of the beam centreline at the west and east face centres of the CV, respectively. $-1 \le \xi \le 1$ is a local parametric coordinate within a CV such that $\xi = -1$ corresponds to face-centre *w* of the west face, $\xi =1$ to the east face centre *e* and $\xi =0$ to the cell-centre ${\textrm{C}}$. The relationship of the arclength parameter $$ \ s \ $$ and the local parameter $$ \ \xi \ $$ is given by, $s = 0.5 [s_w (1-\xi ) + s_e(1+\xi ) ]$, such that the length of the CV, $\textrm{L}_{\textrm{c}} = 0.5(s_e - s_w)$. $s_w$ and $s_e$ are the arclength values of the west and east faces of the CV, respectively. The Hermite splines in Eq. B1 have the following polynomial form,

B2a $$\begin{aligned}&H_r^w (\xi )= \frac{1}{4}(2 \ + \xi )(1 \ - \xi )^2 \ , \nonumber \\ \ {}&H_r^e (\xi ) = \frac{1}{4}(2 \ - \xi )(1 \ + \xi )^2 \end{aligned}$$

B2b $$\begin{aligned}&H_t^w (\xi ) = \frac{1}{4}(1 \ + \xi )(1 \ - \xi )^2 \ , \nonumber \\ \ {}&H_t^e (\xi ) = -\frac{1}{4}(1 \ - \xi )(1 \ + \xi )^2 \end{aligned}$$

To construct a smooth and $C^1-$continuous curve using Hermite splines between a set of points, the coordinates of the set of points as well as the tangent vectors associated with those points, with the condition that the tangents are equal at the points shared by consecutive intervals is required. For constructing the beam centreline curve, the position vectors at the cross-section face centres ($\varvec{r}_w$ and $\varvec{r}_e$) are approximated from the cell-centre position vectors by linear interpolation (Eq. 7). The tangent vectors at the face centres are calculated from the cell-centre position vectors using the central finite difference scheme (Eq. 8). The Hermite spline representation of beam centrelines ensures at least second-order accuracy consistent with the finite volume spatial discretisation. It is used to detect and update the location of the contact points within an iteration of the Newton–Raphson loop where the derivatives of the Eqs. B1 are required. Hence, for completeness, the first and second derivatives of the discretised beam centreline are given by,

B3a $$\begin{aligned}&\varvec{r}_{d,\xi }(\xi ) = H_{r,\xi }^w(\xi ) \ \varvec{r}_w + H_{r,\xi }^e(\xi ) \ \varvec{r}_e + \frac{\Vert \varvec{r}_e - \varvec{r}_w \Vert }{2}\nonumber \\&\qquad \qquad \quad \times \Big [ H_{t,\xi }^w(\xi ) \ \varvec{t}_w + H_{t,\xi }^e(\xi ) \ \varvec{t}_e \Big ] \end{aligned}$$

B3b $$\begin{aligned}&\varvec{r}_{d,\xi \xi }(\xi ) = H_{r,\xi \xi }^w(\xi ) \ \varvec{r}_w + H_{r,\xi \xi }^e(\xi ) \ \varvec{r}_e + \frac{\Vert \varvec{r}_e - \varvec{r}_w \Vert }{2} \nonumber \\&\qquad \qquad \quad \times \Big [H_{t,\xi \xi }^w(\xi ) \ \varvec{t}_w + H_{t,\xi \xi }^e(\xi ) \ \varvec{t}_e \Big ] \end{aligned}$$

where the first and second order derivatives of Hermite splines are represented by $(\cdot )_{,\xi }$ $(\cdot )_{,\xi \xi }$ respectively.

### Remark

The error in predicting the actual beam centreline using Hermite splines depends upon the spacing of the position vectors of the face centres of each control volume of the beam domain and the error in computing the tangent vectors from the position vectors. This error can be reduced using a finer mesh discretisation of the beam domain, especially for an initially curved beam consistent with the spatial discretisation.

## Appendix C: Contact search algorithm

This section describes the contact search procedures adopted for point-to-point and line-to-line contact between a pair of beams.

### Details on point-to-point contact search

To determine the nearest contact point pair $(\xi _c, \bar{\xi }_c)$ between two beams, minimising the two orthogonality conditions (Eq. 15) is necessary. These conditions, generally nonlinear, require linearisation for implementation in a local Newton–Raphson scheme to accurately predict the contact pair for the current beam domain configuration. The linearised equations for $q_1$ and $q_2$ from Eq. 15, originally derived in [17] and represented in matrix form (using $\varvec{r}$ and $\bar{\varvec{r}}$ as shorthand for the beam centrelines $\varvec{r}(\xi ), \bar{\varvec{r}}(\bar{\xi })$), are,

C1 $$\begin{aligned} {[}M] \begin{bmatrix} \Delta \xi \\ \Delta \bar{\xi }\end{bmatrix} =\begin{bmatrix} \left( \varvec{r} - \bar{\varvec{r}}\right) \cdot \varvec{r}_{,\xi } \\ \left( \varvec{r} - \bar{\varvec{r}}\right) \cdot \bar{\varvec{r}}_{,\bar{\xi }} \end{bmatrix} \end{aligned}$$

where [*M*] is the matrix defined in Eq. 23. In each iteration, the contact point updates with the increment $\Delta \xi$, denoted as $\xi _{i+1} = \xi _{i} + \Delta \xi$ (similarly for the other beam). Iterations continue until the absolute value of the increment magnitude falls below a tolerance of $1\times 10^{-4},$ ensuring accuracy to four decimal places. A good initial guess is crucial for Newton–Raphson convergence. Therefore, an initial estimate is established through linear interpolation of the position vector, simplifying Eq. C1 to a set of linear equations. The explicit solution of these equations serves as the starting point for Newton–Raphson iterations. The position vectors assuming linear interpolation and their corresponding derivatives are given by,

C2 $$\begin{aligned} \begin{aligned}&\varvec{r}_d(\xi ) = \frac{1}{2}(1 - \xi ) \varvec{r}_w + \frac{1}{2}(1 + \xi ) \varvec{r}_e \ , \\&\ \varvec{r}_{,\xi } = \frac{1}{2}(\varvec{r}_e - \varvec{r}_w) \ , \\&\varvec{r}_{,\xi \xi } = 0 \varvec{\bar{\varvec{r}}}_d(\bar{\xi }) = \frac{1}{2}(1 - \bar{\xi }) \varvec{\bar{\varvec{r}}}_w + \frac{1}{2}(1 + \xi ) \varvec{\bar{\varvec{r}}}_e \ , \\&\ \varvec{\bar{\varvec{r}}}_{,\bar{\xi }} = \frac{1}{2}(\varvec{\bar{\varvec{r}}}_e - \varvec{\bar{\varvec{r}}}_w) \ , \varvec{\bar{\varvec{r}}}_{,\bar{\xi }\bar{\xi }} = 0 \end{aligned} \end{aligned}$$

Substituting the above equations in the matrix form (Eq. C1) yields the explicit values of $(\xi ,\bar{\xi }) \equiv (\xi _0,\bar{\xi }_0)$ [17] as,

C3 $$\begin{aligned} \begin{aligned} \xi _0 = - (\varvec{\bar{b}} - \varvec{b}) \cdot \frac{\varvec{\bar{t}} (\varvec{\bar{t} \cdot \varvec{t}}) - \varvec{t}(\varvec{\bar{t}} \cdot \varvec{\bar{t}})}{(\varvec{\bar{t}} \cdot \varvec{\bar{t}})(\varvec{t} \cdot \varvec{t}) - (\varvec{\bar{t}} \cdot \varvec{t})^2}\\ \bar{\xi }_0 = (\varvec{\bar{b}} - \varvec{b}) \cdot \frac{\varvec{t} (\varvec{\bar{t} \cdot \varvec{t}}) - \varvec{\bar{t}}(\varvec{t} \cdot \varvec{t})}{(\varvec{\bar{t}} \cdot \varvec{\bar{t}})(\varvec{t} \cdot \varvec{t}) - (\varvec{\bar{t}} \cdot \varvec{t})^2} \end{aligned} \end{aligned}$$

where $\varvec{b} = \varvec{r}_w + \varvec{r}_e$ and $\varvec{t} = \varvec{r}_e - \varvec{r}_w$ (similarly, the values $\varvec{\bar{b}}$ and $\varvec{\bar{t}}$ are defined).

For the point contact search between two discretised beams, the distinction between *master* and *slave* beams is avoided due to the presence of two orthogonality conditions. A linear search is initiated across all cells of one beam to identify potential contact pairs in the neighbouring beam. Considering *m* segments for the first beam ($B_1$ with mean line $\varvec{r}$) and *n* segments for the neighbouring beam ($B_2$ with mean line $\varvec{\bar{\varvec{r}}}$), each segment *i* in $B_1$ undergoes a loop across all segments of $B_2$. In this loop, the initial guess $(\xi _0,\bar{\xi }_0)$ is evaluated (Eq. C3) based on linear interpolation between end faces (Eq. C2). Accepted contact pairs are those where the explicit estimated values fall within $-1 \le (\xi _0,\bar{\xi }_0) \le 1$, rejecting the segment pair (*i*, *j*) if the values lie outside this range. For accepted pairs, $(\xi ,\bar{\xi })$ values are refined per Eq. C1 until convergence, with matrix [*M*] evaluated using the actual Hermite spline interpolation of beam centreline and derivatives (Appendix B).

In a contact simulation, a contact angle limit $\alpha _{\textrm{lim}}$ determines whether the point contact search algorithm or the line-contact search procedure (Sect. C.2) is utilised. A recommended range for $\alpha _{\textrm{lim}}$ is $25^{\circ } - 30^{\circ }$. The initial contact search for a pair of beams involves calculating the contact angle $\alpha _c$ between the tangent vector at the cell centre of segment *i* of the first beam and a segment *j* of the second beam. If $\alpha _c > \alpha _{\textrm{lim}}$, the point contact procedure is employed; otherwise, the line-contact search procedure is initiated.

### Details on line-to-line contact search

The line-to-line contact interaction between beams involves solving a minimal distance problem with one orthogonality condition (Eq. 25). In the master and slave approach, commonly used in numerical contact formulations [7], a biased treatment is applied due to the presence of one orthogonality condition for a pair of beams. The choice of master and slave bodies is influenced by factors such as mesh discretisation coarseness and body rigidity. However, a drawback is that penetration conditions are only checked from the slave body, potentially missing the penetration of the master into the slave surface [7].

To address the bias in the choice of beams in line-to-line contact, a two-half-pass contact approach is adopted, as introduced by Sauer and Lorenzis [42] and utilised by Magliulo et al. [31] for beam-to-beam contact. This method involves performing the contact search twice between a pair of beams by swapping their roles as slave and master. Figure 30 illustrates a typical line contact search between two beams, such as $B_1$ and $B_2$.

In the two-half-pass algorithm for line-to-line contact between beams, during the first pass with $B_1$ as the slave and $B_2$ as the master, the contact force $\varvec{n}{c}$ only affects $B_1$, and during the second pass with roles reversed, the contact force $\varvec{n}^{B_2}{c}$ only affects $B_2$. This prevents over-constraining and ensures an unbiased treatment of contact, deviating from the notion of equal and opposite contact forces on beam bodies. Figure 30 illustrates the process, with cell and face centres denoted as $c^i_j$ and $f^i_j$, respectively. In the first half pass, the shortest distance is determined by projecting $B_1$’s cell centres onto $B_2$’s centroid line. The second half pass involves $B_2$ as the slave, evaluating orthogonal projections on $B_1$ to find the closest contact pairs $(c^2_j, \xi ^2_c)$.

In the two-half-pass approach for line-to-line contact between beams, the determination of possible contact pairs begins with a linear search across all segments. Taking a specific segment in $B_1$ (e.g., with cell centre $c^1_2$), distance vectors $d^1$ and $d^2$ to the end faces ($f^2_2$ and $f^2_3$) of the neighbouring beam $B_2$ are calculated. The product of dot-products, $P \equiv (d^1 \cdot t^2_2)(d^2 \cdot t^2_3)$, is then computed, where $t^2_2$ and $t^2_3$ are tangents at face centres $f^2_2$ and $f^2_3$, respectively. If $P < 0$, indicating the existence of an orthogonal projection on the neighbouring segment ($c^2_2$), the contact pair $(c^1_2, c^2_2)$ is considered; otherwise, it is rejected. A local Newton-Raphson iteration within the segment ($c^2_2$ of $B_2$) is performed to calculate the actual contact point $\xi ^2_c$, continuing until the difference in magnitudes of $\xi ^2_c$ between consecutive iterations falls below a tolerance of $1 \times 10^{-4}$.
